# Generalist phyllosphere taxa dominate microbial communities on macrophytes across a natural salinity gradient

**DOI:** 10.1186/s40793-026-00881-z

**Published:** 2026-04-04

**Authors:** Daniel P. R. Herlemann, David J. Riedinger, Victor Fenández-Juárez, Luis F. Delgado, Anders F. Andersson, Christian Pansch, Lasse Riemann, Mia M. Bengtsson, Greta Gyraite, Thorsten B. H. Reusch, Marija Katarzyte, Sandra Kube, Georg Martin, Marcin Rakowski, Matthias Labrenz

**Affiliations:** 1 Leibniz Institute for Baltic Sea Research Warnemünde (IOW), 18119 Rostock, Germany; 2 Department of Biology, University of Copenhagen, 3000 Helsingør, Denmark; 3 Science for Life Laboratory, Division of Gene Technology, School of Biotechnology, KTH Royal Institute of Technology, 17121 Solna, Sweden; 4 Center for Limnology, Estonian University of Life Sciences, 51013 Tartu, Estonia; 5 Faculty of Science and Engineering, Environmental and Marine Biology, Åbo Akademi University, 20500 Turku/Åbo, Finland; 6 Marine Research Institute, Klaipėda University, 92294 Klaipeda, Lithuania; 7 GEOMAR, Helmholtz-Zentrum für Ozeanforschung Kiel, 24105 Kiel, Germany; 8 Institute of Microbiology, University of Greifswald, 17489 Greifswald, Germany; 9 Estonian Marine Institute, University of Tartu, 12618 Tallinn, Estonia; 10 National Marine Fisheries Research Institute, 81-332 Gdynia, Poland; 11 Present Address: Karolinska Institutet, Department of Microbiology, Tumor and Cell Biology, Stockholm, Sweden

**Keywords:** Seagrass, Baltic Sea, Salty divide, Bacterial community, Protist community

## Abstract

**Background:**

Shallow coastal habitats are characterized by diverse macrophytes and often feature steep abiotic gradients, including salinity variations, which can shape the leaf- surface epi-microbiome (phyllosphere). To elucidate the effect of salinity and host identity on the phyllosphere of aquatic macrophytes in shallow water, we sampled the leaf surface microbiota across a salinity range of 6–15. Samples included the eelgrass *Zostera marina*, as well as the Eurasian water milfoil (*Myriophyllum spicatum*)*,* muskgrass (*Chara* spp*.*), and sago pondweed (*Stuckenia pectinata*) in the brackish Baltic Sea during the summer of 2022. Microbial communities were characterized using 16S and 18S rRNA gene amplicon sequencing.

**Result:**

As hypothesized, the phyllosphere bacterial and protist community composition was distinct from the surrounding seawater microbiome. Typically associated taxa included the genera *Loktanella, Pseudorhodobacter*, the methylotrophic genus *Methylotenera,* unclassified Synechococcales, and Rhodobacteriaceae. Protist genera such as *Picochlorum* were consistently detected across all macrophyte hosts, while *Cocconeis, Cyclotella, Mondous* and unclassified Bacillariophyceae were present in all phyllospheres except *Chara* spp. Both, salinity and host species significantly influenced the composition and prevalence of the microbiota, primarily through shifts in the abundance of typical phyllosphere taxa. However, only 4–11% of phyllosphere taxa were uniquely associated with a specific salinity or macrophyte host.

**Conclusions:**

Our results demonstrate that aquatic macrophytes harbor a distinct and characteristic phyllosphere microbiome. The low proportion of host- or salinity specific taxa suggests that the most abundant members of this community are generalists, broadly adapted to the phyllosphere niche rather than being narrowly specialized. This implies that the presence of the macrophyte itself, providing a stable, nutrient-rich surface, exerts a stronger deterministic influence on the microbial community than the host identity or salinity fluctuations. Consequently, the phyllosphere appears relatively resilient to environmental variability, particularly salinity fluctuations. This highlights the robust nature of host-microbiome interactions and their importance for conservation of aquatic macrophyte ecosystems.

**Supplementary Information:**

The online version contains supplementary material available at 10.1186/s40793-026-00881-z.

## Introduction

Macrophytes colonize extensive regions in shallow coastal zones and play a critical role in maintaining healthy littoral marine ecosystems. These foundation species form dense meadows, transforming otherwise bare, sandy bottoms into complex habitat matrices [1]. Aquatic macrophytes provide valuable ecosystem services, including enhancing water clarity, stabilizing sediment, offering food and shelter for animals, and facilitating nutrient cycling. However, the global biomass of aquatic macrophytes is decreasing at a rate of 2–5% per year, primarily due to human-driven pressures [1, 2]. Recently, efforts to restore and expand aquatic macrophyte meadows have commenced [3]. Understanding macrophyte-associated microbiomes is critical for understanding host health, productivity, and ecosystem function, especially during ecosystem conservation [4, 5].

*Z. marina* is widespread along coastlines throughout the Northern Hemisphere and has been suggested as a model system for aquatic macrophytes [6]. Because the leaves are constantly submerged, microorganisms attached to *Z. marina* interact closely with the surrounding water microbial community, which often serves as source of the surface phyllosphere microbiome. The phyllosphere provides a physical substrate for prokaryotes and eukaryotes with diverse trophic strategies [7] supporting the littoral food web [8]. The phyllosphere microbiome is typically shaped by host species identity [9], spatial factors [10], and local environmental conditions [7]. Contrasting results have been found regarding the presence of the characteristic (“core”) phyllosphere microbiome. While Fahimipour et al. [11] reported that the high variability and spatial turnover depended on the adjacent coastal seawater microbiomes on the bacterial composition of *Z. marina* leaves, other studies have shown that the phyllosphere microbiome is distinct from the microbial communities in the surrounding water and sediment [6, 9, 10, 12–14]. The presence of a characteristic set of microorganisms suggests that aquatic macrophytes maintain intimate ecological interactions with microbial consortia living in association with them. These characteristic microbes, distinct from those of the surrounding microbiome, may be crucial for the macrophytes’s health [4, 5].

Salinity is one of the most important environmental factors structuring bacterial communities globally [15]. Substantial shifts in salinity alter microbial community composition in water columns, sediment, and the bacterial symbionts of aquatic snails [16–18]. A shift between typical freshwater and saltwater species occurs at the salty divide between salinity 7 and 9 [16, 19]. Salinity-driven shifts in the microbial community composition can directly impact functional communities. For example, salinity is the driving factor for the aerobic methanotrophic community [20] and methane emissions [21]. For *Z. marina* the optimal salinity is 10–25 [22]. When salinity drops below 10, both growth and biomass are significantly reduced; however, *Z. marina* is present until salinity 5. In contrast, the Eurasian water milfoil *Myriophyllum spicatum,* the sago pondweed *Stuckenia pectinata*, and the muskgrass *Chara* spp. are primarily freshwater organisms, typically found in environments with salinity levels between 0 and 10 [23–25].

To evaluate the response of the phyllosphere microbiome responds to varying salinity conditions, we investigated the seagrass *Zostera marina* phyllosphere in the Baltic Sea, and compared its microbiome to those of co-occurring macrophytes. We hypothesized that: (1) the phyllosphere represents a unique ecological niche, harboring characteristic microbial taxa distinct from the surrounding seawater; (2) seawater salinity shapes the seagrass microbiome, by altering the abundance of characteristic taxa; and (3) hosts identity further differentiates these communities, with different macrophytes supporting distinct sets of characteristic taxa. To address these hypotheses, the natural salinity gradient of the Baltic Sea [26] provided an ideal system to test these hypothesis.

## Materials and methods

### Sampling

**Fig. 1:**
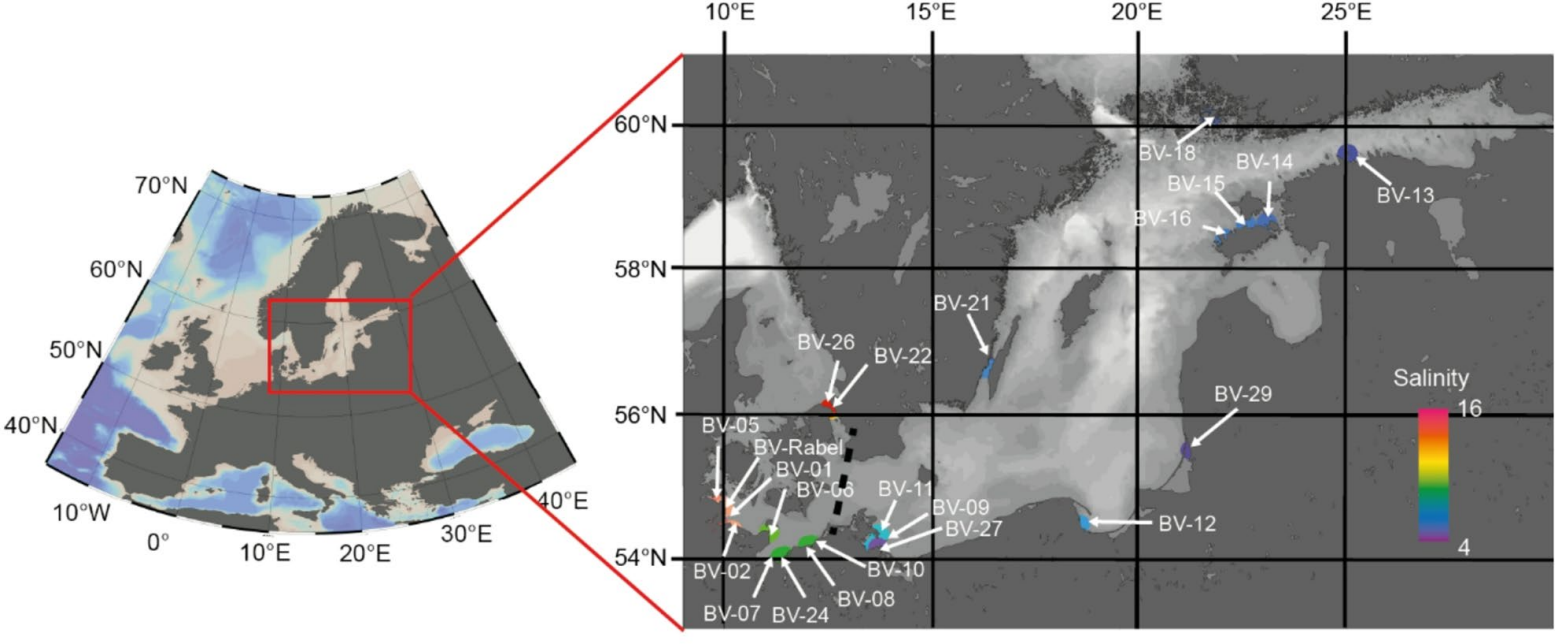
Map of the sampling stations, color-coded by salinity. The dashed black line represents the boundary between areas of differing salinity levels (“salty divide”)

The samples were part of the study by Riedinger et al. [27] and Herlemann et al. [28], however, only those associated with macrophytobenthos were included in this analysis. DNA extraction from both the water column and leaf-epiphytic biofilm (phyllosphere) were retrieved by scuba divers at all designated stations (Table 1, Fig. 1). For water sampling, sterile 100 mL syringes were filled within the meadow. Phyllosphere samples were obtained by cutting leaves at least 20 cm above the sediment. Aquatic macrophytes densities were counted in triplicate 20 × 20 cm squares. Leaf lengths are based on 30 replicates in each meadow. Salinity, temperature (°C), and depth (m) were measured using a CTD48M (Sea and Sun Technology). The dissolved oxygen (mg L^−1^) and pH were measured using HQ40D Portables 2-Channel Multimeter. Phosphate (PO_4_^3−^), nitrate (NO_3_^−^), nitrite (NO_2_^−^), ammonium (NH_4_^+^), and silicate (SiO_2_) concentrations were measured on a Seal Analytical QuAAtro automated constant flow analyzer (SEAL Analytical Ltd. Nordestedt. Germany). Chlorophyll-a (Chl-a) was measured fluorometrically using a 10-AU-005-CE fluorometer (Turner, San Jose, USA).

**Table 1:** Physiochemical parameters at the stations measured (Fig. 1)

	PON	Chla	POC	SiO2	Salinity	Temperature	Sampling depth	Lenght of Zostera	Phyllosphere
Alpha									
BV-01-A	NA	NA	NA	NA	15.0	18.0	NA	NA	*Zostera*
BV-02-A	NA	NA	NA	NA	15.6	17.0	NA	NA	*Zostera*
BV-05-A	2.2	0.7	15.7	NA	15.4	18.2	1.7	59.9	*Zostera*
BV-06-A	3.0	1.0	27.8	5.3	10.6	19.4	4.2	85.4	*Zostera*
BV-07-A	4.2	2.4	23.4	13.1	9.9	21.3	3.2	48.3	*Zostera*
BV-08-A	2.5	0.7	15.1	6.0	9.6	21.4	2.5	74.5	*Zostera*
BV-10-A	3.1	2.3	22.3	5.4	9.3	21.1	3.9	108.8	*Zostera*
BV-22-A	2.7	1.1	21.3	4.1	11.7	17.3	1.5	74.2	*Zostera*
BV-24-A	3.0	1.0	17.5	10.5	9.7	21.1	3.7	38.4	*Zostera*
BV-26-A	2.4	1.5	20.8	3.3	14.0	17.9	1.6	NA	*Zostera*
BV-Rabel-A	NA	NA	NA	NA	15.6	18.0	NA	NA	*Zostera*
	2.9	1.3	20.5	6.8	12.4	19.2	2.8	69.9	
Beta									
BV-09-A	5.0	2.4	37.3	13.0	7.7	20.3	3.7	43.6	*Zostera*
BV-11-A	36.5	20.1	268.5	45.8	7.6	19.8	2.2	52.9	*Zostera*
BV-12-A	3.6	2.1	25.5	6.1	7.0	20.1	2.0	43.8	*Stuckenia*
BV-13-A	5.8	3.6	45.6	6.9	5.5	18.3	2.5		*Stuckenia*
BV-14-A	6.8	2.9	60.8	10.7	6.3	18.5	4.2	38.4	*Zostera*
BV-15-A	4.2	2.2	31.8	12.2	6.6	19.1	1.0		*Stuckenia*
BV-16-A	4.1	1.7	24.6	10.1	6.6	18.2	3.4	41.8	*Zostera*
BV-18-A	6.7	2.1	37.3	11.0	6.1	18.0	3.6	22.5	*Zostera*
BV-21-A	4.2	1.1	20.9	15.0	6.7	16.7	2.6	39.2	*Zostera*
	8.5	4.2	61.4	14.5	6.7	18.8	2.8	40.3	
Oligo									
BV-27-A	53.8	32.9	406.8	88.8	4.3	21.2	1.4		*Myriophyllum*
BV-29-A	NA	18.1	NA	24.6	4.7	19.0	0.7		*Chara*
	53.8	25.5	406.8	56.7	4.5	20.1			

Categorized based on a modified Venice system [64] Alpha, alpha-mesohaline; beta,  beta- mesohaline; oligo,  oligohaline; Chla , Chlorophyl A; DOC , dissolved organic carbon; PON,  particular organic matter; DN , dissolved nitrogen; NA ,  not available

## DNA analysis

To characterize bacterial and eukaryotic communities, triplicate 92 mL water samples were filtered through non-sterile 0.2 µm membrane filters (Merck, Darmstadt, Germany), immediately shock-frozen in liquid nitrogen and stored at − 80 °C. Total genomic DNA was extracted from the whole filter using the DNeasy PowerSoil Pro Kit (Qiagen. Hilden. Germany) according to the manufacturer’s instructions, including a bead-beading step. For the aquatic macrophytes, DNA was extracted using a modified method by Gebbe et al. [29]. Briefly, 4–6 leaf segments (2–3 cm each) were subjected to two rounds of sonication (7 min each) and bead-beaten (30 s at 4 m/s). After the first round of bead-beating the leaf pieces were removed. Subsequently, the manufacturer’s instructions were followed, and the DNA yield was quantified by Picogreen assay (Thermo Fisher. Waltham. USA).

16S rRNA genes were amplified with primers 341F-805R targeting the prokaryotic V3–V4 region [16], and 18S rRNA genes with primers V4F-V4RB targeting the eukaryotic V4 region [30], with the same PCR protocols as in Latz et al. [31]. The primers were supplemented with lllumina sequencing adapters in the 5’ ends. For 16S rRNA gene, both the forward and reverse primers were phased to increase the complexity of the library while for 18S rRNA, only the forward primer was phased [31]. PCR products were purified using the MagSi-NGS PREP Plus Kit (MDKT00010075, magtivio BV, Nuth, the Netherlands). The purified products were indexed through a second PCR following the Adapterama I indexing scheme [32]. Amplicons were multiplexed and sequenced using the Illumina MiSeq sequencing platform (Illumina Inc. San Diego. CA. US) by SciLifeLab/NGI (Solna. Sweden).

Phased primer sequences were removed from the reads using a Snakemake pipeline (https://github.com/biodiversitydata-se/amplicon-multi-cutadapt). The workflow included removal of read-pairs that contain Illumina adapters, exclusion of read-pairs lacking the expected 5’ primer sequences, trimming of the primer sequences from the retained reads, and elimination of read pairs with incorrectly positioned primer. DADA2 was used for denoising (truncLen = 250, minLen = 150, maxEE = 2), merging paired-end reads, and chimera removal. Amplicon sequence variants (ASVs) were taxonomically assigned with DADA2 [33] using PR2 (V. 4.14.0) [34] as a reference database for 18S rRNA gene amplicons and Silva 138.1 for 16S rRNA gene amplicons [35]. ASVs were aggregated based on identical taxonomic assignment at the highest possible resolution (“taxa”). Non-target sequences including Archaea, mitochondria, chloroplast, Metazoa, and Streptophyta were removed prior to downstream analysis. Streptophyta, encompassing the macrophyte taxa sampled in this study often represented the majority of the 18S rRNA gene phyllosphere samples, and their exclusion significantly decreased the final sequence depth.

## Statistical analysis

Statistics were performed in R-studio [36] using the *vegan* package [37] and PAST [38]. Sequences assigned to the 16S rRNA gene were rarefied to 8720 reads per sample, and those assigned to the 18S rRNA gene were rarefied to 1561 reads per sample after removing all the non-target reads. Rarefaction curve analysis suggests sufficient depth and adequacy of sequencing in a sample after rarefaction (SFig. 7, SFig. 9). Venn Diagrams were generated using InteractiVenn [39] based on presence/absence of sequences, where a relative overlap between samples was also calculated.

A Kruskal–Wallis test followed by a post hoc Tukey’s pairwise test was used to calculate significant differences between the numbers of taxa between samples. The dataset was partitioned by substrate to independently assess the influence of environmental factors on each community. Similarity Percentages (SIMPER) analysis was then employed to identify the species driving dissimilarities between groups. To ensure ecological relevance, we only reported species that exhibited at least a two-fold difference in mean abundance between groups and a relative abundance > 1%.

The effects of environmental variables on the betadiversity pattern across the samples from water, sediment, and biofilm were analyzed by PERMANOVA (Permutational Multivariate Analysis of Variance) tests and displayed using Principal Coordinate Analysis (PCoA), both based on Bray–Curtis dissimilarity using the rarefied dataset. The number of permutations was set to 9999.

## Results

### Characterization of the sampling site

The relative bacterial community composition (16S rRNA gene amplicons) was analyzed from 57 water and 78 phyllosphere samples, while the eukaryotic community composition (18S rRNA gene amplicons) was analyzed from 71 water samples and 74 phyllosphere samples. The sampling sites included stations in the alpha-mesohaline region (salinity 15.6–9.3), specifically BV-01, BV-02, BV-05, BV-06, BV-07, BV-08, BV-10, BV-22, BV-24, BV-26, and BV-Rabel, where only *Z. marina* was sampled (Table 1, Fig. 1). The beta-mesohaline region (salinity 7.8–6.0) included stations BV-09, BV-11, BV-12, BV-13, BV-14, BV-15, BV-16, BV-18, and BV-21, where both *Z. marina* and *S. pectinata* were present. The oligohaline region (salinity 4.3–4.7) included BV-27 and BV-29 were *M. spicatum* and *Chara* spp. were sampled. A Tukey’s pairwise test showed that, in addition to salinity, *Z. marina* leaf length was significantly different between the alpha-mesohaline and beta-mesohaline regions (Tukey test, *p* < 0.01). Temperature, chlorophyll A, and SiO_2_ concentrations in the oligohaline region differed significantly from those in the mesohaline regions. No significant differences were detected for sampling depth, particulate organic carbon (POC), and particulate organic nitrogen (PON) between the alpha-mesohaline and beta-mesohaline regions (*p* > 0.01). Data from the oligohaline stations were incomplete and, therefore, not included in these analyses.

## Bacterial community composition at different salinities and for different host species

**Fig. 2:**
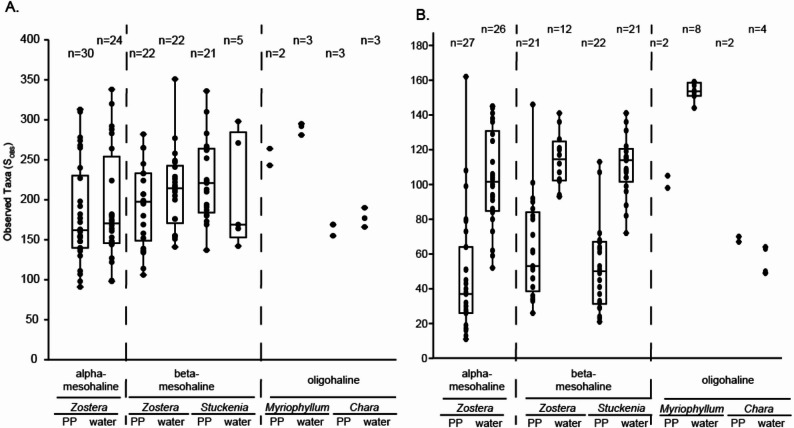
Boxplot of observed taxa (S_OBS_) of the **A** bacterial community and **B** microeukaryotic community under alpha-, beta-mesohaline and oligohaline condition. PP = phyllosphere

The relative number of observed taxa (S_OBS_), i.e., richness, exhibited high variability within both the phyllosphere and water bacterial community across different host species and salinity regions (Fig. 2A). Although a Kruskal–Wallis test suggested a significant differences between the samples (*p* = 0.02); post-hoc Tukey’s pairwise test revealed no significant differences between individual groups (*p* > 0.05).

**Fig. 3:**
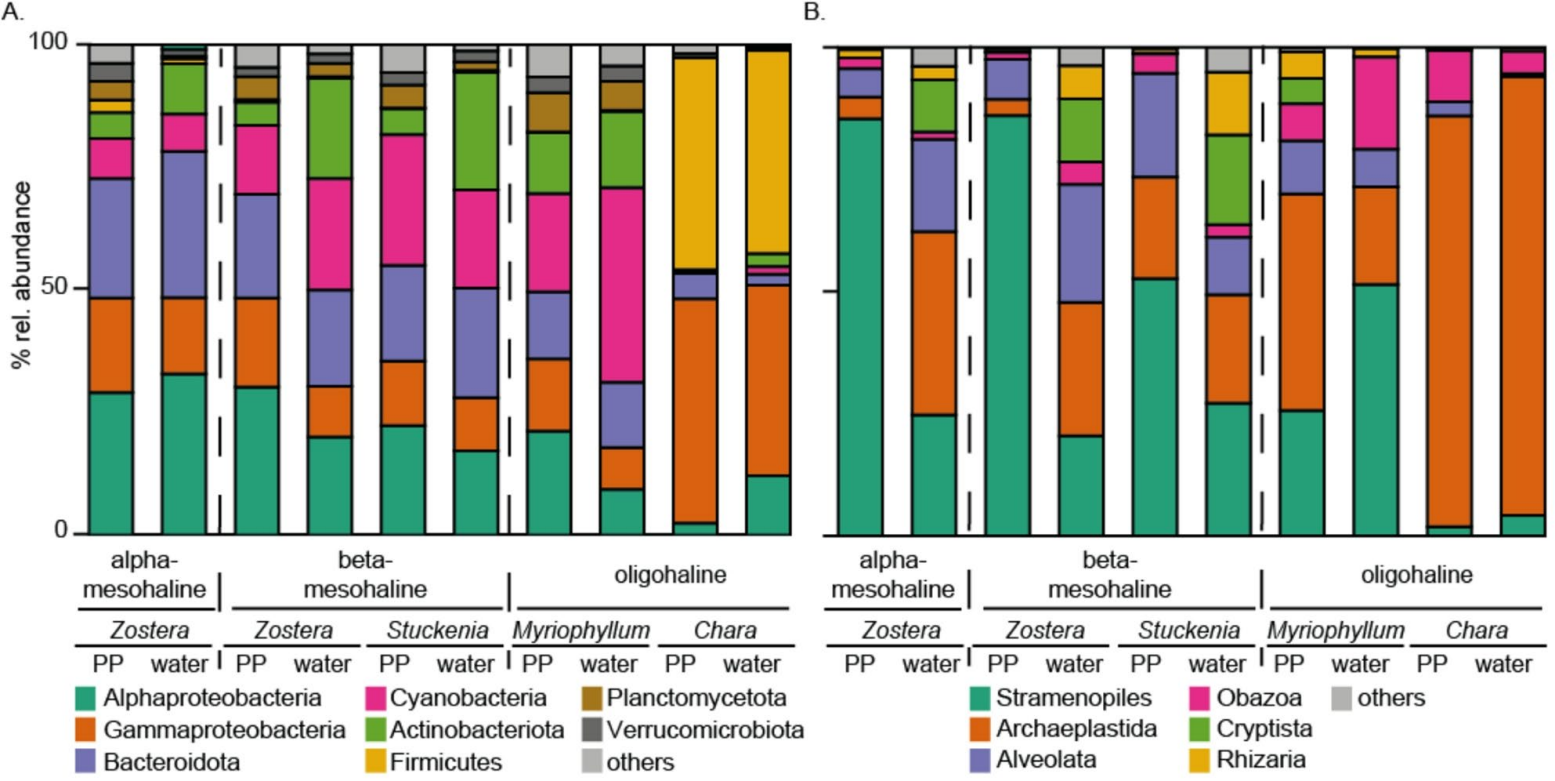
Stacked bar graph of **A** bacterial and **B** microeukaryotic phyla/classes relative (% rel.) abundances at alpha-, beta-mesohaline and oligohaline condition. Phyla/classes < 1% are summarized as "other". PP = phyllosphere

The most abundant phyla/classes based on relative abundance of the bacterial communities across all samples were Alphaproteobacteria (3–33%), Gammaproteobacteria (9–46%), Bacteroidota (2–30%), Cyanobacteria (0–40%), and Actinobacteria (1–24%; Fig. 3A). An exception was observed at the *Chara* spp. station (BV-29, Curonian Lagoon), where Gammaproteobacteria (phyllosphere 39%, water 46%) and Firmicutes (phyllosphere 41%, water 43%) dominated, while other phyla/classes were almost absent.

Only *Z. marina* phyllosphere samples were obtained from stations representing alpha-mesohaline and beta-mesohaline conditions. These samples exhibit high abundances of Alphaproteobacteria (29% alphamesohaline, 30% betamesohaline) and, Gammaproteobacteria (19% alphamesohaline, 18% betamesohaline), with relatively stable composition across the salty divide. In contrast, these classes declined in the water column upon crossing the salty divide (Alphaproteobacteria: 33% alphamesohaline, 20% betamesohaline; Gammaproteobacteria: 16% alphamesohaline, 10% betamesohaline). The water column generally contained higher relative abundances of Cyanobacteria, Actinobacteriota and Planktomycetota compared with the phyllosphere.

**Fig. 4:**
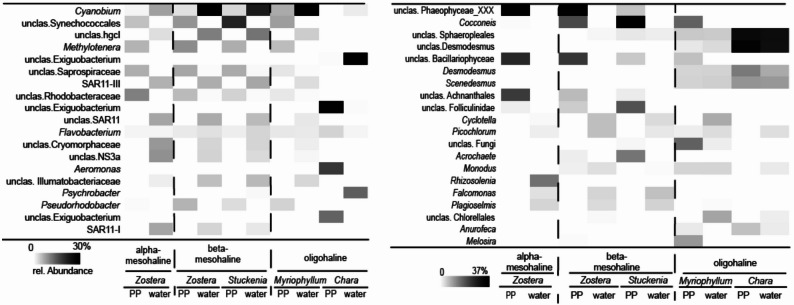
Heatmap of dominant **A** bacterial and **B** microeukaryotic taxa at alpha-, beta-mesohaline and oligohaline condition (one ASV per row). Only taxa with an average relative abundance at of > 1% are included. PP = phyllosphere. unclas. = unclassified

At a finer taxonomic level, distinct separation between phyllosphere and the water microbiome became evident (Fig. 4A). The most abundant taxa, based on relative abundance, included the cyanobacterial *Cyanobium* (0.2–28.8%) and unclassified Synechococcus (0–15.4%). *Cyanobium* was significantly more abundant in the water column compared to unclassified Synechococcales (Mood’s Test *p* = 0.01, S Table 1). Additional abundant phyllosphere-associated taxa included unclassified Saprospiraceae (0–6.3%), unclassified Rhodobacteriaceae (0–8.9%), *Methylotenera* (0–8.4%) and *Pseudorhodobacter* (0–5.3%). The taxa assigned to *Flavobacterium* (0.7–3.2%) was the only abundant taxon present in both the water and the phyllosphere. With the exception of station BV-29, the water column bacterial community was consistent with typical Baltic Sea communities, including hgcI (0.10%), SAR11 clade (0–6.3%, and 0–6.6%), unclassified Cryomorphaceae (0–7.3%), NS3a (0–7.8%), and unclassified Illumatobacteriaceae (0–5.1%) [16].

At station BV-29, the *Chara* spp. phyllosphere was dominated by members of the genus *Exiguobacterium*. Finer-scale taxonomic analysis revealed pronounced shifts in the relative abundance between the water and phyllosphere (SFig 1). The most abundant bacteria on the *Chara* spp. phyllosphere were relatives of *Exiguobacterium acetylicum* (19.6%)*, E. sibiricum* (11.1%)*, Aeromonas allosaccharophila* (13.7%) and *Acinetobacter bouvetii* (1.6%). In the water column, relatives of *Exiguobacterium aestuarii* (11.0–22.5%) and *E. oxidotolerans* (4.7–14.7%) were dominant, along with high abundances of *Psychrobacter faecalis* (9.3–12.4%) (Gammaproteobacteria).

**Fig. 5:**
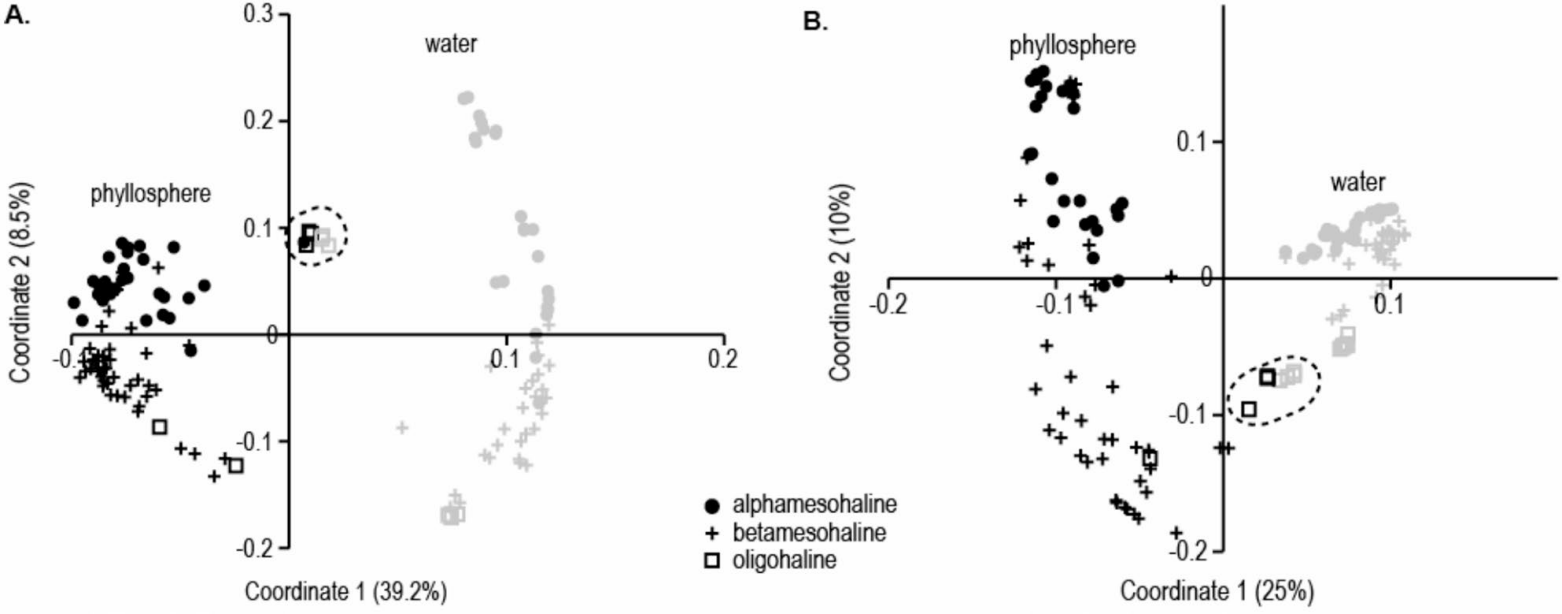
Principle Coordinate Analysis of **A** bacterial (16S) and **B** microeukaryotic (18S) rRNA gene community composition of water (gray) and phyllosphere (black). Symbols indicate the different salinity regions: alphamesohaline (dot), betamesohaline (+ ) and oligohaline (square). The oligohaline samples from station BV29 are circumscribed with a dotted line

Principal Coordinate Analysis (PCoA) of the bacterial community composition revealed a difference between the water and phyllosphere bacterial community along the first coordinate, which accounted for most of the variation (38.2% variation; one-way PERMANOVA: all samples: *R*^*2*^ = 37%, F = 80.2, *p* < 0.01; Fig. 5 A, detailed PERMANOVA in Table 2). Differences between the alpha- and beta-mesohaline and oligohaline regions were separated along the second coordinate (8.5% variation; two-way PERMANOVA: substrate F = 106.1, *p* < 0.01; salinity F = 14.0, *p* < 0.01; interaction F = 9.7, *p* < 0.01). Samples from the *Chara* spp. station (BV-29) were distinctly separated from the other samples (Fig. 5A dotted line).

**Table 2:** One-way PERMANOVA test comparing differences in the community composition between water and phyllosphere

	Alphamesohaline			Betamesohaline			Oligohaline		
	F-value	*R*^*2*^	*p* value	F-value	*R*^*2*^	*p* value	F-value	*R*^*2*^	*p* value
Bacteria	49.5	48.8%	< 0.01	81.0	53.9%	< 0.01	3.0	25.2%	0.02
Protists	31.2	37.9%	< 0.01	37.7	33.7%	< 0.01	3.8	21.4%	0.01

Investigating only the *Z. marina* phyllosphere bacterial communities between alpha and beta-mesohaline regions also indicated a significant shift in the phyllosphere bacterial community in response to salinity (one-way PERMANOVA: salinity *R*^*2*^ = 12% F = 6.7, *p* < 0.01; Table 3, SFig. 2). Additionally, the bacterial community composition in the water column shifted significantly between the alpha- and beta-mesohaline regions (one-way PERMANOVA: *R*^*2*^ = 29%, F = 17.8, *p* < 0.01; Table 3), consistent with previous observations [28]. Within the oligohaline region, the phyllosphere bacterial community differed significantly from that of the surrounding water (one-way PERMANOVA: *R*^*2*^ = 25%, F = 3.0, *p* = 0.02; Table 2).

**Table 3:** One-way PERMANOVA test comparing the bacterial community composition between alpha- and beta-mesohaline conditions (salinity) and between *S. pectinata* and *Z. marina* (host)

	Salinity			Host		
	F-value	*R*^*2*^	*p* value	F-value	*R*^*2*^	*p* value
Phyllosphere	6.7	11.8%	< 0.01	4.9	10.5%	< 0.01
Water	17.8	28.8%	< 0.01	1.3	5%	0.21

**Fig. 6:**
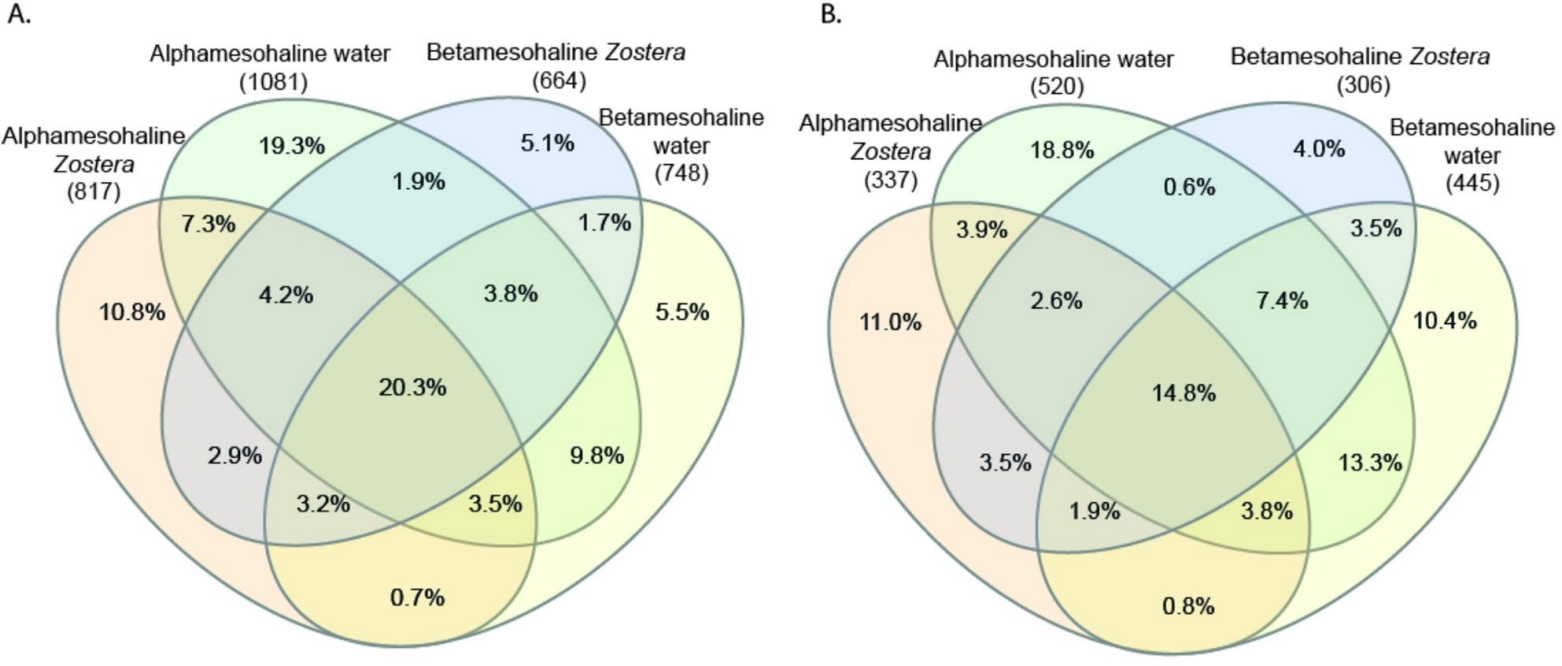
Venn diagram displaying overlaps in **A** bacterial and **B** microeukaryotic community composition of the *Zostera marina* phyllosphere and water community at different salinities. Shown are the percentages of the total community (all taxa)

SIMPER identified the species that contributed most to the dissimilarities between the salinity regions. Among the abundant taxa *Methylotenera*, *Pseudorhodobacter*, *Synechococcus* and *Cyanobium* (Fig. 4**, **STable 2) were more characteristic of the beta-mesohaline regions. In contrast, unclassified Rhodobacteriaceae, unclassified Saprospiraceae, *Yoonia*-*Loktanella*, *Perspicuibacter,* and *Portibacter* were more abundant in the alpha-mesohaline bacterial community. Despite the differences between the water and phyllosphere microbiome, the phyllosphere and the water microbiomes shared 35.3% of the taxa under alpha-mesohaline conditions and 29% at beta-mesohaline conditions (Fig. 6). Across all four sample types, 20.3% of the taxa were commonly present. A total of 30.6% of the taxa (473 taxa) were found in all *Z. marina* phyllosphere samples, and the overlap between the alpha- and beta-mesohaline water taxa was 30.2%.

Investigating different phyllosphere bacterial community under beta-mesohaline condition revealed a significant difference between *Z. marina* and *S. pectinata* (Table 3, one-way PERMANOVA: host *R*^*2*^ = 11% F = 4.9, *p* < 0.01; SFig. 3). Interestingly, the water bacterial community composition did not significantly differ between the stations where *Z. marina* and *S. pectinata* were sampled (one-way PERMANOVA: Table 3, host *R*^*2*^ = 5% F = 1.3, *p* = 0.21).

SIMPER analysis indicated that unclassified Synechococcales, *Phormidesmis* and unclassified Caldilineaceae were more characteristic of *S. pectinata*, whereas *Pseudorhodobacter, Yoonia-Loktanella*, *Symbiobacter, Methylotenera* and unclassified Pirellulaceae were more prominent in *Z. marina* (Stable 3). Under beta-mesohaline conditions, the overlap between the *S. pectinata* and *Z. marina* phyllosphere was 41.2% and the overlap between the water samples from the *Z. marina* and *S. pectinata* meadows was 43.9%. The *S. pectinata* phyllosphere and water shared 25.5% of the taxa (SFig 6).

## Protist community composition at different salinities and host species

In contrast to the bacterial community, a significant difference in the relative numbers of taxa (S_OBS_) was detected between the phyllosphere and water protists community (Fig. 2B; Kruskal–Wallis *p* < 0.01). In the mesohaline region, the number of protists taxa (S_OBS_) was significantly higher in water column than on the phyllosphere (Tukey’s *p* < 0.01). Under oligohaline conditions, the protists taxa (S_OBS_) were highest at the *M. spicatum-*station (BV-27) and lowest at the *Chara* spp. station (BV-29). However, these patterns should be interpreted with caution due to the limited number of samples available from these stations.

The protist communities in the phyllosphere were primarily composed of Stramenopiles (2–86%), Archaeplastida (3–84%), Alveolata (2–21%) and Obazoa (2–19%) (Fig. 3B). In addition to Stramenopiles, Alveolata, and Archaeplastida, protists in the water were affiliated with Rhizaria and Cryptista. With the exception of BV-29, Stramenopiles had a higher relative abundance in the phyllosphere (51–86%) compared to water (20–27%), whereas Archaeplastida were more abundant in the water (22–44% vs. phyllosphere 4–21%). There were also clear differences in the abundance of the phyla and classes across the salty divide. Similar to the bacterial communities, the major phyla remained relatively constant on the phyllosphere (Stramenopiles: alphamesohaline = 85%, betamesohaline = 86%, Archaeplastida: alphamesohaline = 4%, betamesohaline = 3%, Alveolata: alphamesohaline = 6%, betamesohaline = 8%) but changed in the water column (Stramenopiles: alphamesohaline = 25%, betamesohaline = 20%, Archaeplastida: alphamesohaline = 27%, betamesohaline = 21%, Alveolata: alphamesohaline = 24%, betamesohaline = 21%). Compared to the other investigated host phyllosphere microbiomes, the Obazoa abundance was highest (19%) in the *M. spicatum* phyllosphere, and Archaeplastida abundance was highest (84%) on the *Chara* spp. phyllosphere.

On finer taxonomic level, several unclassified taxa that were not present in the corresponding water sample were abundant on *Z. marina* and *S. pectinata* but absent on *M. spicatum* (Fig. 4B). This included unclassified Phaeophyceae, unclassified Bacillariophyceae, unclassified Achnanthales and unclassified Foluculinidae. The situation was different on *M. spicatum* and *Chara* spp., where unclassified Sphaeropleales (5–23%), unclassified *Desmodesmus* (2–24%), *Desmodesmus* (4–10%), *Monodus* (0–4%)*,* and *Scenedesmus* (4–10%) were abundant on the phyllosphere and in the water samples. *Cocconeis* was not found on the alphamesohaline *Z. marina* phyllosphere samples but at the betamesohaline conditions of *Z. marina* and *S. pectinata* phyllospheres, as well as on the oligohaline *M. spicatum* phyllospheres.

Similar to the bacterial communities, the protist communities were separated between the water and phyllosphere along the first PCoA axis, which explained the highest variation in the dataset. Protist communities across the salinity regions were separated along the second coordinate (two-way PERMANOVA: substrate F = 59.8, *p* < 0.01; salinity F = 16.4, *p* < 0.01; interaction F = 6.9, *p* < 0.01, Fig. 5B). Additionally, the protist community sampled at the *Chara* spp.*-*station BV-29 was distinctly separated from the other oligohaline samples.

Analyzing the impact of the salinity shift on the *Z. marina* phyllosphere microbiome between alpha- and beta-mesohaline conditions revealed a significant change in the protist community (Table 4, one-way PERMANOVA: salinity *R*^*2*^ = 13%, F = 6.6, *p* < 0.01; SFig. 4). Under beta-mesohaline condition, the protist’s community associated with *Z. marina* and *S. pectinata* also showed a significant difference (Table 4, one-way PERMANOVA: *R*^*2*^ = 14%, F = 6.7, *p* < 0.01; SFig. 5). Furthermore, the water protist community differed significantly between the hosts (one-way PERMANOVA: substrate *R*^*2*^ = 13%, F = 4.5, *p* < 0.01). This was also the case under oligohaline conditions, where the water and phyllosphere protists microbiome were significantly different (Table 4).

**Table 4:** One-way PERMANOVA test comparing the protists community composition between alpha- and betamesohaline conditions (salinity) and between *S. pectinata* and *Z. marina* (host)

	Salinity			Host		
	F-value	*R*^*2*^	*p* value	F-value	*R*^*2*^	*p *value
Phyllosphere	6.6	13.0%	< 0.01	6.7	14.1%	< 0.01
Water	7.0	13.4%	< 0.01	4.5	12.9%	< 0.01

Despite the major differences between water and the *Z. marina* phyllosphere, these environments shared 25.1% of taxa under alpha-mesohaline conditions and 27.6% under beta-mesohaline conditions (Fig. 6B). The water protist community samples shared 39.3% of the taxa between alpha- and beta-mesohaline conditions. A total of 22.8% of the taxa overlapped between the alpha-and beta-mesohaline *Z. marina* phyllosphere samples. Under beta-mesohaline conditions, the overlap between the *S. pectinata* and *Z. marina* phyllosphere community was 30.9% of taxa, and the overlap between water samples from the *Z. marina* and *S. pectinata* meadows was 38.9% of taxa. The *S. pectinata* phyllosphere and water shared 27.0% of the taxa (SFig 6B).

SIMPER analysis indicated that unclassified Phaeophyceae_XXX, *Cocconeis*, *Acrochaete,* and unclassified Folliculinidae_X were characteristic of the phyllosphere (Stable 4). Unclassified Phaeophyceae_XXX were characteristic for alpha-mesohaline conditions, while *Cocconeis* was *Chara* spp. for beta-mesohaline conditions (Stable 5). Both *Cocconeis* and unclassified Phaeophyceae_XXX were present at beta-mesohaline conditions, but *Cocconeis* was more typical for *M. spicatum*, and unclassified Phaeophyceae_XXX was more typical for *Z. marina* (Stable 6).

## Discussion

In this study, we compared the phyllosphere of aquatic macrophytes with the surrounding water microbiome across different salinity regions and host species. The microbiomes of the water and the phyllosphere were significantly different, consistent with findings from several previous studies [7, 10, 40, 9] (Figs. 4 and 5). Despite these clear differences between habitats, nearly one-third of the bacterial and eukaryotic taxa were shared (Fig. 6).

Aquatic macrophytes are in constant contact with the surrounding water, making seawater a natural source of microbial inoculation. However, the phyllosphere is not a passive reflection of the water microbiome, it is actively shaped by host-specific selection, environmental filtering and macrophyte-microbe interaction. As demonstrated by Vogel et al. [41] both host identity and environmental conditions play key roles in structuring the phyllosphere, with only a subset of water-derived microbes successfully colonizing macrophytes surfaces. The phyllosphere selects for specific microorganisms through its unique surface chemistry and exudates, resulting in a characteristic microbial community composition. These characteristic taxa were previously found in the phyllosphere of macrophytes [7, 42], highlighting the role of host-driven selection. Among the most abundant taxa was an unclassified Synechococcales. Although primarily known for their phototrophic free-living lifestyle, *Synechococcales* have been documented in symbiotic association with corals [43] and have been found attached to *Z. marina* in the Baltic Sea [7]. Their presence on macrophyte surfaces suggest potential ecological roles in nutrient cycling or host protection, though the functional significance of this association remains unclear. The abundant genus *Methylotenera* is frequently associated with the *Z. marina* phyllosphere across different studies [7, 40, 42]. These methylotrophs are known to oxidize methanol and formaldehyde exuded as a waste product from the leaves. Furthermore, they may contribute to methane production through the degradation of methylophsphonate components [44, 45]. The abundant unclassified Saprospiraceae and unclassified Rhodobacteriaceae have also been described as characteristic of the aquatic phyllosphere and are potentially involved in the degradation of complex organic compounds from dead tissue [7, 40, 46].

Interestingly, many of these characteristic and abundant taxa were also found on artificial seagrass leaves in previous studies [42], suggesting that they do not rely exclusively on macrophyte surfaces and are rather generalist in biofilm production. In contrast to the large number of generalists phyllosphere taxa, relatively few taxa were host-specific. When comparing the bacterial sequences comprising the *Z. marina* and *S. pectinata* phyllosphere*,* only 10% of the taxa were specific to one species, whereas most taxa were present in both, with relatively equal contributions in the SIMPER analysis (Stable 1). More sequences per sample may had increased the number of overlapping taxa since rarer taxa are currently incompletely covered and the overlap may be underestimated. Therefore, specialized phyllosphere taxa seem to be relatively rare. The presence of phyllosphere generalists may also explain the stability of the bacterial community during shifts in salinity compared to the water-bacterial community (Fig. 3).

Similar to the bacterial biofilm, macrophyte leaves serve as a stable habitat for marine diatoms and other algae. Interestingly, the protist community of *Z. marina* was mostly composed of Stramenopiles, Alveolata, and Rhizaria in contrast to water, where Crypista and Obozoa were also abundant. Diatoms (Stramenopiles; Bacillariaceae) commonly represent the largest component of the microscopic epiphytic biomass associated with seagrass leaves [47] and are considered to be part of *Z. marina*’s “core” phyllosphere community [7, 48]. Based on 18S rRNA gene analysis, previous studies described several characteristic phyllosphere genera, such as *Prorocentrum, Amphidinium,* Peronosporomycetes clade II, and *Anisolpidium* [48]*.* These genera were either low in abundance or absent in our study (0–0.6%). However, these studies were conducted under seawater conditions. Under brackish conditions in the Baltic Sea, high abundances of Cocconeidaceae, Bacillariaceae and Ectocarpales on *Z. marina* leaves were identified [7]. These groups were also present in our study; however, finer taxonomic resolution will be necessary to determine if the taxa of the studies are similar.

The protist genus *Cocconeis* is described as a typical freshwater diatom commonly associated with algae and macrophytes [49]. Its absence under high salinity conditions of the alpha-mesohaline zone in our study is consistent with its known freshwater affinity. In contrast, the unclassified Phaeophyceae, (Phaeophyceae_XXX), which was a characteristic taxon in the *Z. marina* phyllosphere belongs to the brown algae that are typically not symbiotic, multicellular organisms. Although Phaeophyceae are not known to form symbiotic associations with seagrasses, their presence in our and previous studies [7] suggests that cell fragments or detritus may settle on the *Z. marina* shoots. The pathogenic protist *Labyrinthula zosterae* (“wasting disease”), a known causative agent of *Zostera* die-off [50], was detected at low abundance in a single water sample from the “Rabel” station. This finding, while limited in occurrence, highlights the potential presence of disease-related taxa in the study area, warranting further monitoring.

## Impact of salinity

A study on the global distribution of bacterial diversity, marked salinity as the most important driver of bacterial community composition, surpassing the effects of temperature and pH [15]. Similarly, in the Baltic Sea, salinity has the strongest influence on microbial communities [16, 18, 51, 52]. The most significant shift in the biota composition occurs at the salty divide between alpha-mesohaline and beta-mesohaline conditions (“horohalinicum”: salinity 7–9), which was also the focus of our study. We found that the microbial community on the phyllosphere across the salty divide differed significantly (Tables 3**, **4). The changes in the microbial community in both water and phyllosphere were primarily due to changes in the abundance of the characteristic taxa (Fig. 4). Only 5.1% of the beta-mesohaline taxa and 10.8% of the alpha-mesohaline bacterial taxa were specific to the phyllosphere in their respective salinities (Fig. 6), indicating that over 90% of the taxa were still present at different salinities.

Changes in the phyllosphere microbiome in response to environmental conditions are well documented [7, 53, 54, 55]. However, in addition to abiotic environmental factors, host-related factors such as seagrass productivity also drive the phyllosphere microbiome [7, 28]. The shoot leaf area has been identified as determinant of the phyllosphere bacterial community in the Baltic Sea [7]. Accordingly, changes in seagrass leaf length in our study could also be responsible for shifts in the microbial community. Previous studies suggested that shifts in the taxonomic composition often occur within the framework of functional redundancy, where essential metabolic functions are maintained by other taxa [14]. Our observation of a relatively constant number of observed taxa (S_OBS_) across salinity regions and hosts (Fig. 2) supports this concept, suggesting that the overall richness is preserved despite community turnover. This pattern was expected for the bacterial community, as prior studies demonstrated that bacterial richness remains stable across at changing salinities, including within host-associated microbiomes [17, 56, 57]. Regarding protists, previous research reported both decreased [51], and an increased alphadiversity [52] across the brackish salinity transition. Consistent with other molecular-based studies [58], the protist communities in this study maintained a relatively constant number of taxa (Fig. 2). However, while the alphadiversity remained constant, significant taxonomic turnover occurred in response to changing salinity levels.

## Macrophyte host effects at oligohaline conditions

Differences between the water and phyllosphere microbiota at oligohaline conditions were significant (Table 2). However, the oligohaline samples consisted of relatively few samples, with major differences in composition between the *M. spicatum* and *Chara* spp*.* microbiome. Phyllosphere samples from *M. spicatum* followed the general trends observed in other phyllospheres, supporting the presence of characteristic bacterial community taxa. This suggests that despite local variability, a shared microbial signature persists across macrophyte hosts in low salinity environments. However, the *Chara* spp*.* phyllosphere samples differed from all other samples even at a broad phylogenetic level (Fig. 3). The most abundant bacteria on the *Chara* spp*.* phyllosphere were assigned to firmicutal *Exiguobacterium*, a genus known for its tolerance to extreme environmental conditions, including fluctuating oxygen, temperatures and salinity levels [59]. Phylogenetic assignment of the unclassified Exiguobacterium revealed close affiliations with known species lineages (SFig 1). *Exiguobacterium aestuarii* and *E. oxidotolerans* were highly abundant in the water column, along with *Psychrobacter facealis* (Gammaproteobacteria). In contrast, *E. acetylicum* was predominantly associated with *Chara* spp. phyllosphere. *E. acetylicum* has been described as a macrophyte associated bacterium capable of degrading a variety of organic compounds and tolerating moderate salinity [60]. In contrast, *E. aestuarii* was originally isolated from tidal flats and is well adapted to estuarine environments, exhibiting high tolerance to salinity fluctuations [61]. Therefore, a specific phyllosphere bacterial community was also found on the *Chara* spp*.* phyllosphere samples. The presence of abundant *Exiguobacterium* at BV-29 could be due to the presence of *Chara* spp*.,* which develops small rhizoids rather than leaves, or specific local conditions at BV-29. The sampling site BV-29 is a sheltered lagoon close to an industrial harbor and channel system. Since both the water and *Chara* spp. phyllosphere consisted of high abundances of *Exiguobacterium*, while previous studies identified members of the Cytophaga-Flavobacteria-Bacterioidetes as abundant on Baltic Sea *Chara* spp*.* [62], the presence of *Exiguobacterium* seems to be related to specific environmental conditions at the sampling site. *Exiguobacterium* are possibly derived from the nearby canal system where they have previously been found [63]. A strong wind a day before sampling may have mixed and transported those bacteria to the sampling site. Therefore, we assume that the observed phyllosphere bacterial community is rather untypical for *Chara* spp*.* meadows and related to storm events. This also indicates that the phyllosphere microbiome can be influenced by local conditions under extreme conditions.

The most abundant taxa of the protist phyllosphere community of *Chara* spp*.* were unclassified Sphaeropleales and Desmodesmus, belonging to the Chlorophyceae, which are commonly found in freshwater environments. However, these Chlorophyceae are usually not found to be host-associated but settle on sediment during periods of low water movement. Therefore, these Chlorophyceae could have been transported by the storm before sampling and possibly settled on the phyllosphere. However, unclassified Sphaeropleales and Desmodesmus were also found on the leaf and water of *M. spicatum* but in lower abundance (Fig. 4). In contrast to the Chlorophyceae, the presence of *Cocconeis* and unclassified Bacilariophyceae on the *M. spicatum* leaves suggests that these may represent characteristic general phyllosphere protists. Conversely, *Anurofeca* was only present in the oligohaline samples of *M. spicatum* and *Chara* spp., while *Melosira* was found exclusively on *M. spicatum,* suggesting a higher degree of host specify. Given the scarcity of research on protist community assembly in oligohaline macrophyte habitats, it remains to be determined which of the protists constitute a characteristic phyllosphere microbiome.

## Conclusion

Our results support the hypothesis that the phyllosphere of aquatic macrophytes consists of a suite of characteristic bacterial and protist taxa where many are likely involved in key ecological functions, including the utilization of complex carbohydrates and metabolic by-products (e.g., methanol) derived from macrophyte leaves. An exclusive host or salinity-specific microbiome, was only apparent for a small subset of taxa. Instead, the effect of salinity and host species was primarily characterized by shifts in the abundance of the general phyllosphere-associated taxa.

These taxa seem to have characteristics allowing them to form biofilms on the leaves of multiple macrophyte species suggesting a relatively unspecific interaction with the host. This implies that the general physiological and structural characteristics of the host macrophytes are more important for microbial colonization than species specific traits.

The resilience of the phyllosphere to small salinity fluctuations further underscores the stability of macrophyte–microbiome interactions under moderate environmental change. This stability may be particularly relevant for seagrass conservation efforts, where maintaining microbial associations could enhance macrophyte survival and ecosystem recovery. However, extreme environmental events, such as the storm observed at the *Chara* spp. station can disrupt the phyllosphere, leading to the temporary dominance of opportunistic taxa. This highlights the vulnerability of the phyllosphere to acute disturbances, and its dependence on the surrounding water column as a source of microbial communities. Together, these findings emphasize that host-microbiome stability is a critical component of aquatic macrophytes resilience.

## Supplementary Information

Below is the link to the electronic supplementary material.

Supplementary Material 1.

Supplementary Material 2.

## Data Availability

The datasets supporting the conclusions of this article are available in the European Nucleotide Archive under the accession number PRJEB68222 in compliance with the Minimal Information about any (X) Sequence (MIxS) standard through the brokerage service GFBio93. Environmental data are available at IOWMeta (doi.io-warnemuende.de/10.12754/data-2023-0010). Additional information is available at https://datacloud.io-warnemuende.de/s/tLGkFp4sb8DzoF4 and https://owncloud.io-warnemuende.de/index.php/s/Lg5dEqX0g3NctJm

## References

[1] Duarte CM. The future of seagrass meadows. Environ Conserv. 2002;29(2):192–206.

[2] Orth RJ, Carruthers TJ, Dennison WC, Duarte CM, Fourqurean JW, Heck KL, et al. A global crisis for seagrass ecosystems. Bioscience. 2006;56(12):987–96.

[3] Kraufvelin P, Olsson J, Bergström U, Bryhn A, Bergström L. Restoration measures for coastal habitats in the Baltic Sea: cost-efficiency and areas of highest significance and need. 2021.

[4] Berendsen RL, Pieterse CM, Bakker PA. The rhizosphere microbiome and plant health. Trends Plant Sci. 2012;17(8):478–86. 22564542 10.1016/j.tplants.2012.04.001

[5] Laforest-Lapointe I, Paquette A, Messier C, Kembel SW. Leaf bacterial diversity mediates plant diversity and ecosystem function relationships. Nature. 2017;546(7656):145–7. 28538736 10.1038/nature22399

[6] Ettinger CL, Voerman SE, Lang JM, Stachowicz JJ, Eisen JA. Microbial communities in sediment from *Zostera marina* patches, but not the Z. *marina* leaf or root microbiomes, vary in relation to distance from patch edge. PeerJ. 2017;5:e3246. 28462046 10.7717/peerj.3246 PMC5410140

[7] Bengtsson MM, Bühler A, Brauer A, Dahlke S, Schubert H, Blindow I. Eelgrass leaf surface microbiomes are locally variable and highly correlated with epibiotic eukaryotes. Front Microbiol. 2017;8:1312. 28751881 10.3389/fmicb.2017.01312 PMC5507959

[8] Duffy JE, Reynolds PL, Boström C, Coyer JA, Cusson M, Donadi S, et al. Biodiversity mediates top–down control in eelgrass ecosystems: a global comparative-experimental approach. Ecol Lett. 2015;18(7):696–705. 25983129 10.1111/ele.12448

[9] Crump BC, Wojahn JM, Tomas F, Mueller RS. Metatranscriptomics and amplicon sequencing reveal mutualisms in seagrass microbiomes. Front Microbiol. 2018;9:388. 29599758 10.3389/fmicb.2018.00388 PMC5863793

[10] Ugarelli K, Chakrabarti S, Laas P, Stingl U. The seagrass holobiont and its microbiome. Microorganisms. 2017;5(4):81. 29244764 10.3390/microorganisms5040081 PMC5748590

[11] Fahimipour AK, Kardish MR, Lang JM, Green JL, Eisen JA, Stachowicz JJ. Global-scale structure of the eelgrass microbiome. Appl Environ Microbiol. 2017;83(12):e03391-e3416. 28411219 10.1128/AEM.03391-16 PMC5452814

[12] Hurtado-McCormick V, Kahlke T, Petrou K, Jeffries T, Ralph PJ, Seymour JR. Regional and microenvironmental scale characterization of the *Zostera muelleri* seagrass microbiome. Front Microbiol. 2019;10:1011. 31139163 10.3389/fmicb.2019.01011 PMC6527750

[13] Mejia AY, Rotini A, Lacasella F, Bookman R, Thaller MC, Shem-Tov R, et al. Assessing the ecological status of seagrasses using morphology, biochemical descriptors and microbial community analyses. A study in *Halophila stipulacea* (Forsk.) Aschers meadows in the northern Red Sea. Ecol Indic. 2016;60:1150–63.

[14] Roth‐Schulze AJ, Zozaya‐Valdés E, Steinberg PD, Thomas T. Partitioning of functional and taxonomic diversity in surface‐associated microbial communities. Environ Microbiol. 2016;18(12):4391–402. 27062175 10.1111/1462-2920.13325

[15] Lozupone CA, Knight R. Global patterns in bacterial diversity. Proc Natl Acad Sci U S A. 2007;104(27):11436–40. 17592124 10.1073/pnas.0611525104 PMC2040916

[16] Herlemann DP, Labrenz M, Jürgens K, Bertilsson S, Waniek JJ, Andersson AF. Transitions in bacterial communities along the 2000 km salinity gradient of the Baltic Sea. ISME J. 2011;5(10):1571–9. 10.1038/ismej.2011.41. 21472016 10.1038/ismej.2011.41 PMC3176514

[17] Kivistik C, Knobloch J, Käiro K, Tammert H, Kisand V, Hildebrandt J-P, et al. Impact of salinity on the gastrointestinal bacterial community of *Theodoxus fluviatilis*. Front Microbiol. 2020;11:683. 32457702 10.3389/fmicb.2020.00683 PMC7225522

[18] Klier J, Dellwig O, Leipe T, Jürgens K, Herlemann DP. Benthic bacterial community composition in the oligohaline-marine transition of surface sediments in the Baltic Sea based on rRNA analysis. Front Microbiol. 2018;9:236. 29520255 10.3389/fmicb.2018.00236 PMC5827536

[19] Remane A. Die Brackwasserfauna. Verh Dtsch Zool Ges. 1934;36:34–74.

[20] Deng Y, Liu Y, Dumont M, Conrad R. Salinity affects the composition of the aerobic methanotroph community in alkaline lake sediments from the Tibetan Plateau. Microb Ecol. 2017;73:101–10. 27878346 10.1007/s00248-016-0879-5

[21] Soued C, Bogard MJ, Finlay K, Bortolotti LE, Leavitt PR, Badiou P, et al. Salinity causes widespread restriction of methane emissions from small inland waters. Nat Commun. 2024;15(1):717. 38267478 10.1038/s41467-024-44715-3 PMC10808391

[22] Nejrup LB, Pedersen MF. Effects of salinity and water temperature on the ecological performance of *Zostera marina*. Aquat Bot. 2008;88(3):239–46.

[23] Hillmann E, DeMarco K, La Peyre M. Submerged aquatic vegetation and environmental data along a salinity gradient in Barataria Bay, Louisiana (2015). US geological survey data release. 2017. 10.5066/F7M61HG4.2017

[24] Blindow I, Schütte M. Elongation and mat formation of *Chara aspera* under different light and salinity conditions. Hydrobiologia. 2007;584(1):69–76. 10.1007/s10750-007-0578-9.

[25] Teeter JW. Effects of sodium chloride on the sago pondweed. J Wildl Manage. 1965. 10.2307/3798562.

[26] Reissmann JH, Burchard H, Feistel R, Hagen E, Lass HU, Mohrholz V, et al. Vertical mixing in the Baltic Sea and consequences for eutrophication–A review. Prog Oceanogr. 2009;82(1):47–80.

[27] Riedinger DJ, Fernández-Juárez V, Delgado LF, Sperlea T, Hassenrück C, Herlemann DP, et al. Control of *Vibrio vulnificus* proliferation in the Baltic Sea through eutrophication and algal bloom management. Commun Earth Environ. 2024;5(1):246.

[28] Herlemann DP, Delgado LF, Riedinger DJ, Fernández-Juárez V, Andersson AF, Pansch C, et al. Low impact of *Zostera marina* meadows on sediment and water microbiota under brackish conditions. Environ Microbiome. 2025;20(1):2. 39799374 10.1186/s40793-024-00662-6 PMC11724437

[29] Gebbe R, Kesy K, Hallier D, Brauer A, Bertilsson S, Labrenz M, et al. Ecology of potentially pathogenic *Vibrio* spp. in a seagrass meadow ecosystem. Aquat Microb Ecol. 2025;91:15–30.

[30] Balzano S, Abs E, Leterme SC. Protist diversity along a salinity gradient in a coastal lagoon. Aquat Microb Ecol. 2015;74:263–77.

[31] Latz MA, Andersson A, Brugel S, Hedblom M, Jurdzinski KT, Karlson B, et al. A comprehensive dataset on spatiotemporal variation of microbial plankton communities in the Baltic Sea. Sci Data. 2024;11(1):18. 38168085 10.1038/s41597-023-02825-5 PMC10761891

[32] Glenn TC, Nilsen RA, Kieran TJ, Sanders JG, Bayona-Vásquez NJ, Finger JW, et al. Adapterama I: universal stubs and primers for 384 unique dual-indexed or 147,456 combinatorially-indexed Illumina libraries (iTru & iNext). PeerJ. 2019;7:e7755. 31616586 10.7717/peerj.7755 PMC6791352

[33] Callahan BJ, McMurdie PJ, Rosen MJ, Han AW, Johnson AJA, Holmes SP. DADA2: high-resolution sample inference from Illumina amplicon data. Nat Methods. 2016;13(7):581–3. 27214047 10.1038/nmeth.3869 PMC4927377

[34] Guillou L, Bachar D, Audic S, Bass D, Berney C, Bittner L, et al. The Protist Ribosomal Reference database (PR2): a catalog of unicellular eukaryote small sub-unit rRNA sequences with curated taxonomy. Nucleic Acids Res. 2012;41(D1):D597–604. 23193267 10.1093/nar/gks1160 PMC3531120

[35] Quast C, Pruesse E, Yilmaz P, Gerken J, Schweer T, Yarza P, et al. The SILVA ribosomal RNA gene database project: improved data processing and web-based tools. Nucleic Acids Res. 2012;41(D1):D590–6. 10.1093/nar/gks1219. 23193283 10.1093/nar/gks1219 PMC3531112

[36] Studio R. Integrated development environment for R. Boston, MA: R Studio Inc.; 2018.

[37] Dixon P. VEGAN, a package of R functions for community ecology. J Veg Sci. 2003;14(6):927–30.

[38] Hammer Ø, Harper DA. Past: paleontological statistics software package for educaton and data anlysis. Palaeontol Electron. 2001;4(1):1.

[39] Heberle H, Meirelles GV, da Silva FR, Telles GP, Minghim R. InteractiVenn: a web-based tool for the analysis of sets through Venn diagrams. BMC Bioinformatics. 2015;16:1–7. 25994840 10.1186/s12859-015-0611-3 PMC4455604

[40] Sanders-Smith R, Segovia BT, Forbes C, Hessing-Lewis M, Morien E, Lemay MA, et al. Host-specificity and core taxa of seagrass leaf microbiome identified across tissue age and geographical regions. Front Ecol Evol. 2020;8:605304.

[41] Vogel MA, Mason OU, Miller TE. Host and environmental determinants of microbial community structure in the marine phyllosphere. PLoS ONE. 2020;15(7):e0235441. 32614866 10.1371/journal.pone.0235441 PMC7332025

[42] Adamczyk EM, O’Connor MI, Parfrey LW. Seagrass (*Zostera marina*) transplant experiment reveals core microbiota and resistance to environmental change. Mol Ecol. 2022;31(19):5107–23. 35933734 10.1111/mec.16641

[43] Meunier V, Bonnet S, Pernice M, Benavides M, Lorrain A, Grosso O, et al. Bleaching forces coral’s heterotrophy on diazotrophs and *Synechococcus*. ISME J. 2019;13(11):2882–6. 10.1038/s41396-019-0456-2. 31249389 10.1038/s41396-019-0456-2 PMC6794269

[44] Kalyuhznaya MG, Martens‐Habbena W, Wang T, Hackett M, Stolyar SM, Stahl DA, et al. Methylophilaceae link methanol oxidation to denitrification in freshwater lake sediment as suggested by stable isotope probing and pure culture analysis. Environ Microbiol Rep. 2009;1(5):385–92. 23765891 10.1111/j.1758-2229.2009.00046.x

[45] Li S, Dong X, Humez P, Borecki J, Birks J, McClain C, et al. Proteomic evidence for aerobic methane production in groundwater by methylotrophic *Methylotenera*. ISME J. 2025. 10.1093/ismejo/wraf024. 39927982 10.1093/ismejo/wraf024 PMC11978286

[46] Papazachariou V, Fernández-Juárez V, Parfrey LW, Riemann L. Nitrogen fixation and microbial communities associated with decomposing seagrass leaves in temperate coastal waters. Microb Ecol. 2024;87(1):106. 39141097 10.1007/s00248-024-02424-w PMC11324715

[47] Prazukin AV, Lee RI, Firsov YK, Kapranov SV. Vertical distribution of epiphytic diatoms in relation to the eelgrass *Zostera noltii* canopy biomass and height. Aquat Bot. 2022;176:103466.

[48] Trevizan Segovia B, Sanders‐Smith R, Adamczyk EM, Forbes C, Hessing‐Lewis M, O’Connor MI, et al. Microeukaryotic communities associated with the seagrass *Zostera marina* are spatially structured. J Eukaryot Microbiol. 2021;68(1):e12827. 33065761 10.1111/jeu.12827

[49] GUIRY MD. AlgaeBase. World-wide electronic publication, National university of Ireland, Galway. 2010. http://www.algaebase.org.

[50] Muehlstein LK, Porter D, Short FT. *Labyrinthula zosterae* sp. nov., the causative agent of wasting disease of eelgrass, *Zostera marina*. Mycologia. 1991;83(2):180–91.

[51] Olli K, Ptacnik R, Klais R, Tamminen T. Phytoplankton species richness along coastal and estuarine salinity continua. Am Nat. 2019;194(2):E41–51. 31318279 10.1086/703657

[52] Telesh IV, Schubert H, Skarlato SO. Revisiting Remane’s concept: evidence for high plankton diversity and a protistan species maximum in the horohalinicum of the Baltic Sea. Mar Ecol Prog Ser. 2011;421:1–11.

[53] Crump BC, Koch EW. Attached bacterial populations shared by four species of aquatic angiosperms. Appl Environ Microbiol. 2008;74(19):5948–57. 18676705 10.1128/AEM.00952-08 PMC2565956

[54] Webb SJ, Rabsatt T, Erazo N, Bowman JS. Impacts of Zostera eelgrasses on microbial community structure in San Diego coastal waters. Elem Sci Anth. 2019;7:11.

[55] Li X, Wang H, Zang Y, Xue S, Xin J, Liu L, et al. Exploring the structure and assembly of seagrass microbial communities in rhizosphere and phyllosphere. Appl Environ Microbiol. 2025;91(3):e02437-e2524. 10.1128/aem.02437-24. 39992122 10.1128/aem.02437-24 PMC11921323

[56] Kivistik C, Käiro K, Tammert H, Sokolova IM, Kisand V, Herlemann DP. Distinct stages of the intestinal bacterial community of *Ampullaceana balthica* after salinization. Front Microbiol. 2022;13:767334. 36110301 10.3389/fmicb.2022.767334 PMC9468257

[57] Van der Loos LM, D’hondtEngelenPaviaTothWillems SAHHGBA, et al. Salinity and host drive Ulva-associated bacterial communities across the Atlantic-Baltic Sea gradient. Mol Ecol. 2023;32(23):6260–77. 35395701 10.1111/mec.16462

[58] Hu YO, Karlson B, Charvet S, Andersson AF. Diversity of pico-to mesoplankton along the 2000 km salinity gradient of the Baltic Sea. Front Microbiol. 2016;7:679. 27242706 10.3389/fmicb.2016.00679 PMC4864665

[59] Vishnivetskaya TA, Kathariou S, Tiedje JM. The *Exiguobacterium* genus: biodiversity and biogeography. Extremophiles. 2009;13:541–55. 19381755 10.1007/s00792-009-0243-5

[60] Farrow JA, Wallbanks S, Collins MD. Phylogenetic interrelationships of round-spore-forming bacilli containing cell walls based on lysine and the non-spore-forming genera *Caryophanon*, *Exiguobacterium*, *Kurthia*, and *Planococcus*. Int J Syst Evol Microbiol. 1994;44(1):74–82. 10.1099/00207713-44-1-74 8123563

[61] Kim I-G, Lee M-H, Jung S-Y, Song JJ, Oh T-K, Yoon J-H. *Exiguobacterium aestuarii* sp. nov. and *Exiguobacterium marinum* sp. nov., isolated from a tidal flat of the Yellow Sea in Korea. Int J Syst Evol Microbiol. 2005;55(2):885–9. 15774680 10.1099/ijs.0.63308-0

[62] Hempel M, Blume M, Blindow I, Gross EM. Epiphytic bacterial community composition on two common submerged macrophytes in brackish water and freshwater. BMC Microbiol. 2008;8:1–10. 18402668 10.1186/1471-2180-8-58 PMC2386815

[63] Gyraite G, Kataržytė M, Overlingė D, Vaičiūtė D, Jonikaitė E, Schernewski G. Skip the dip—avoid the risk? Integrated microbiological water quality assessment in the South-Eastern Baltic Sea coastal waters. Water Basel. 2020;12(11):3146.

[64] den Hartog C. Typologie des Brackwassers. Helgol Wiss Meeresunters. 1964;10(1):377–90.

