# Supplementary material for: Generalist phyllosphere taxa dominate microbial communities on macrophytes across a natural salinity gradient
Source: Environ Microbiome. 2026 Apr 4;21:52. doi: 10.1186/s40793-026-00881-z (PMC13067490; doi:10.1186/s40793-026-00881-z)
Supplement: Supplementary file 1 — Supplementary Material 1. [file 40793_2026_881_MOESM1_ESM.pdf]

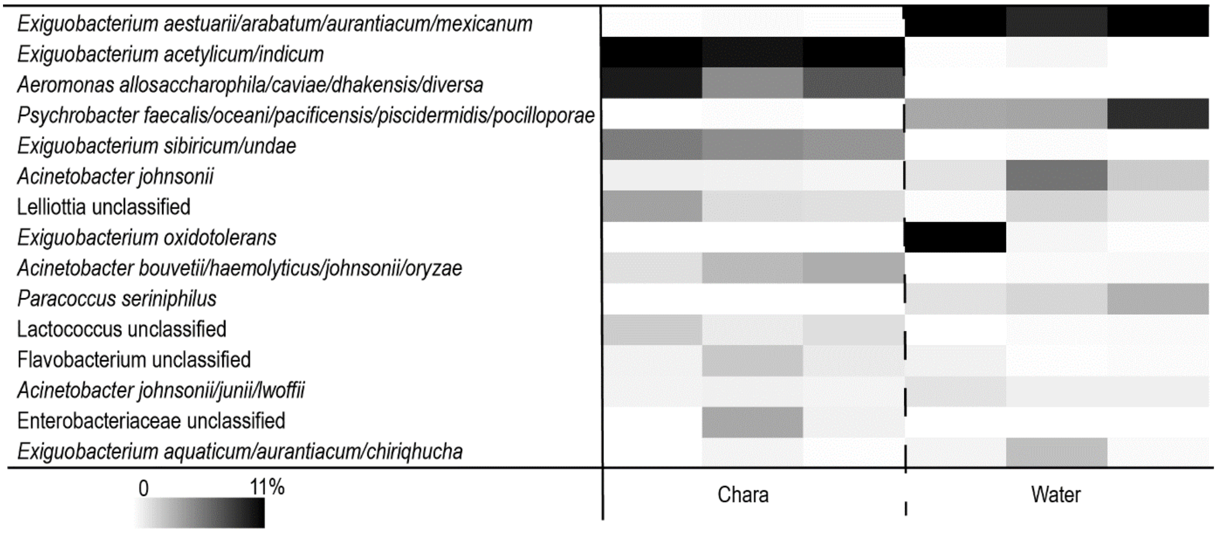

SFig 1. (A) Bacterial community composition on the *Chara* spp. phyllosphere and the water phyllosphere on finest possible taxonomic resolution. Taxa can be assigned to various species within a phylogenetic lineage as indicated in the different species names in the table.

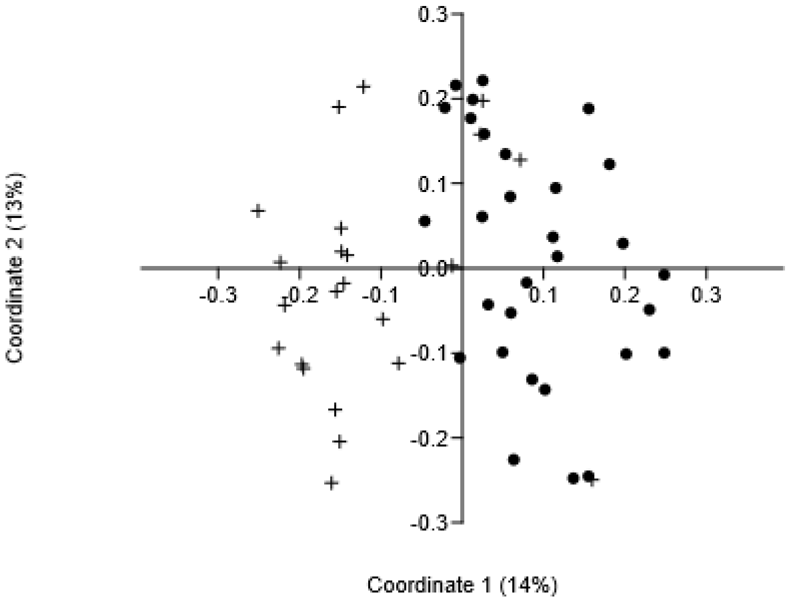

SFig 2. PCoA anlysis of alpha (dot) -and beta (plus)-mesohaline bacterial community composition on the *Zostera marina* phyllosphere.

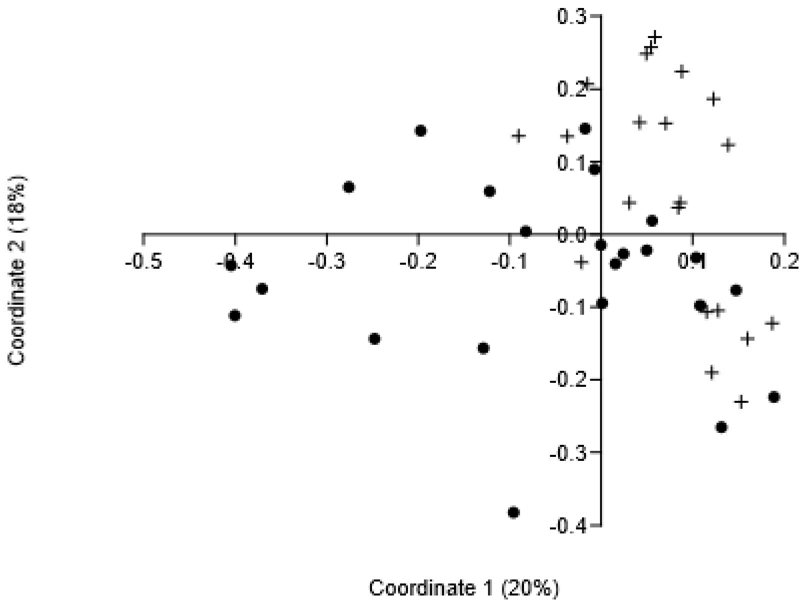

SFig 3. PCoA anlysis of *Stuckneia* (dot) -and *Zostera marina* (plus) phyllosphere bacterial community composition.

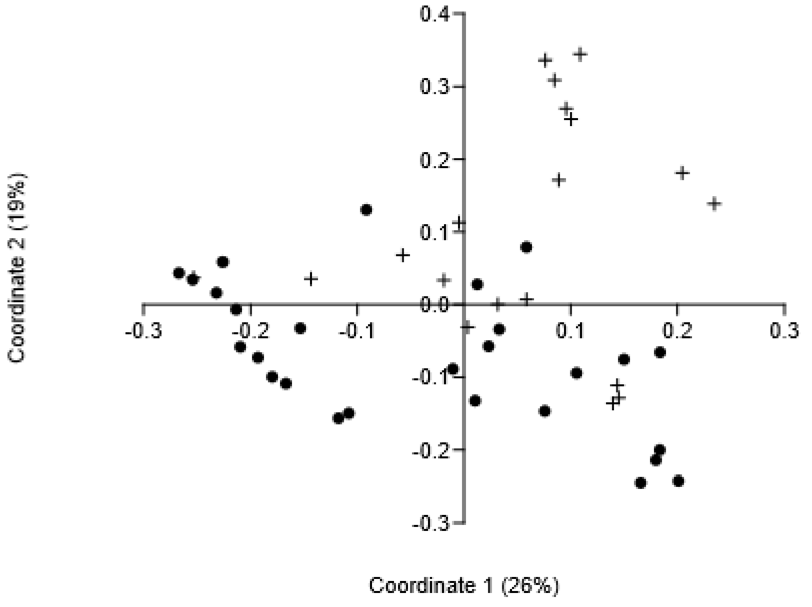

SFig 4. PCoA anlysis of alpha (dot) -and beta (plus)-mesohaline protist community composition on the *Zostera marina* phyllosphere.

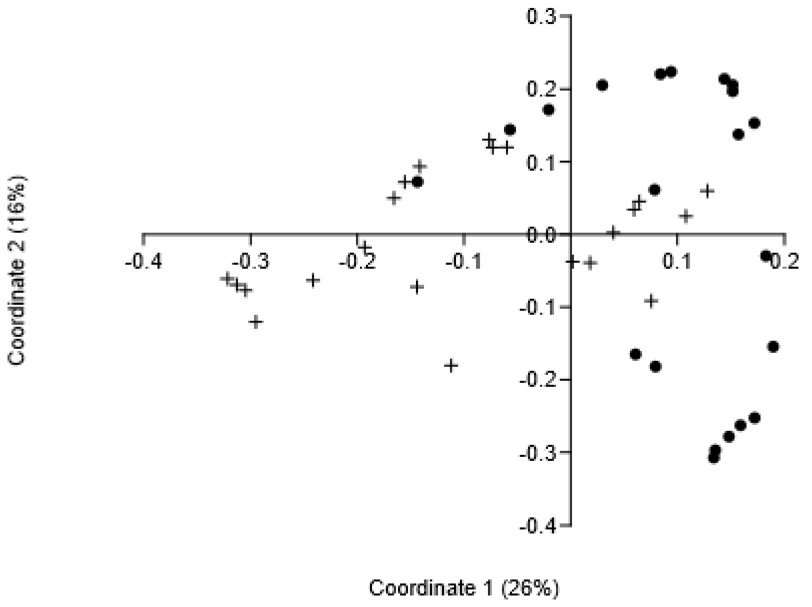

SFig 5. PCoA anlysis of *Stuckneia* (dot) -and *Zostera marina* (plus) phyllosphere protist community composition.

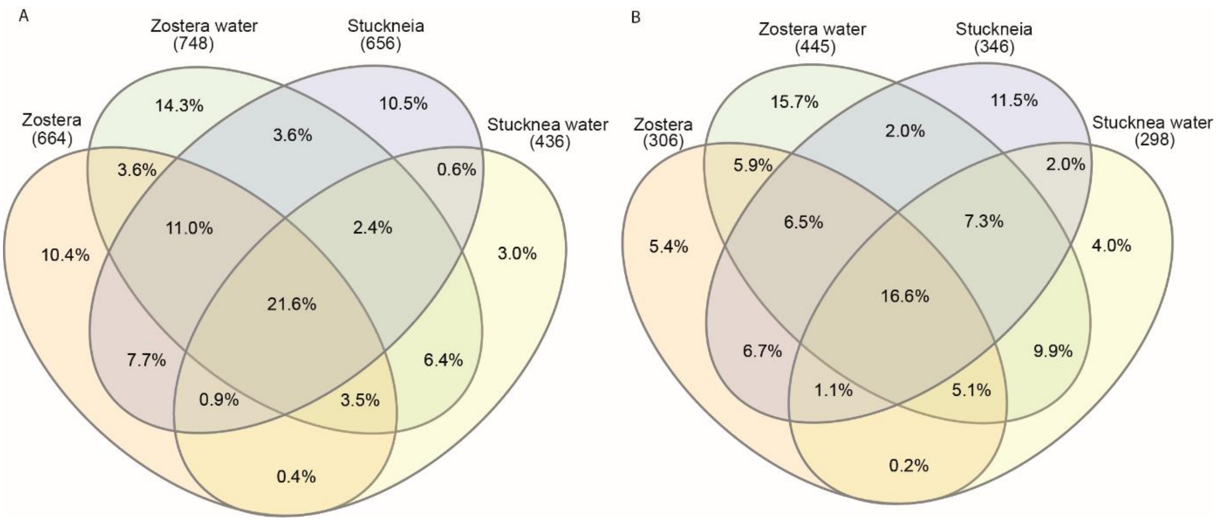

SFig 6. Venn Diagram of the (A) bacterial community and (B) microeukaryotic community composition of phyllosphere and water community at different host species. Shown are percentages of the total community.

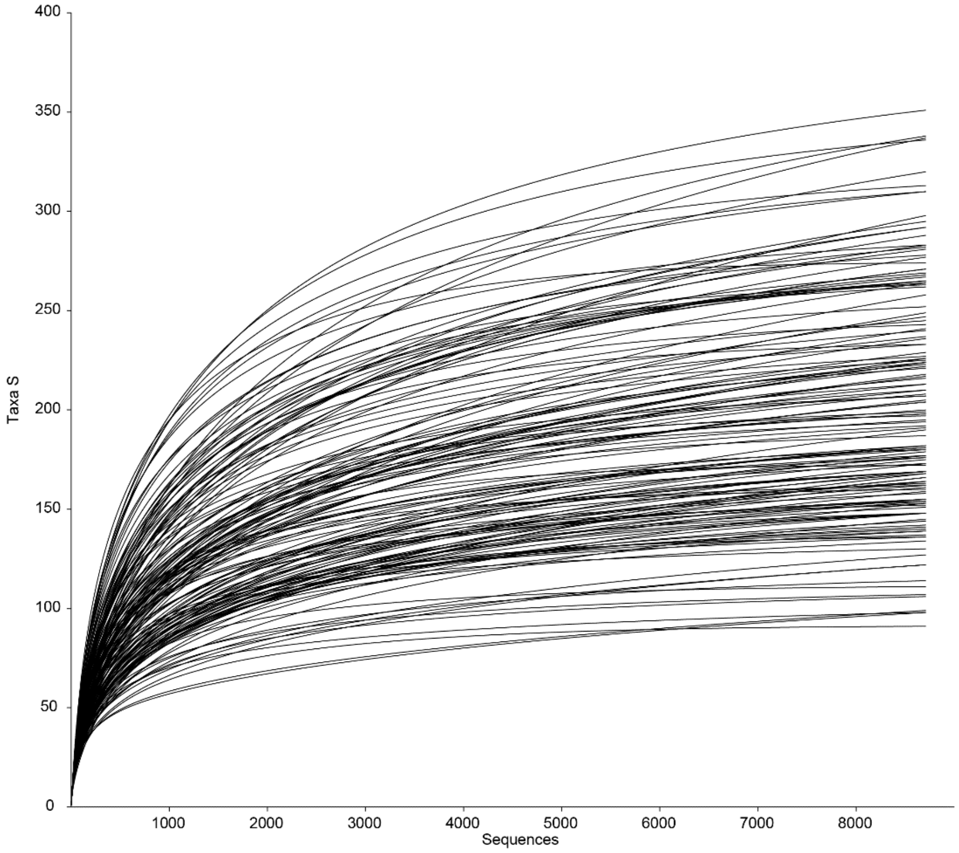

SFig 7. Rarefaction curve of the rarefied bacterial communities.

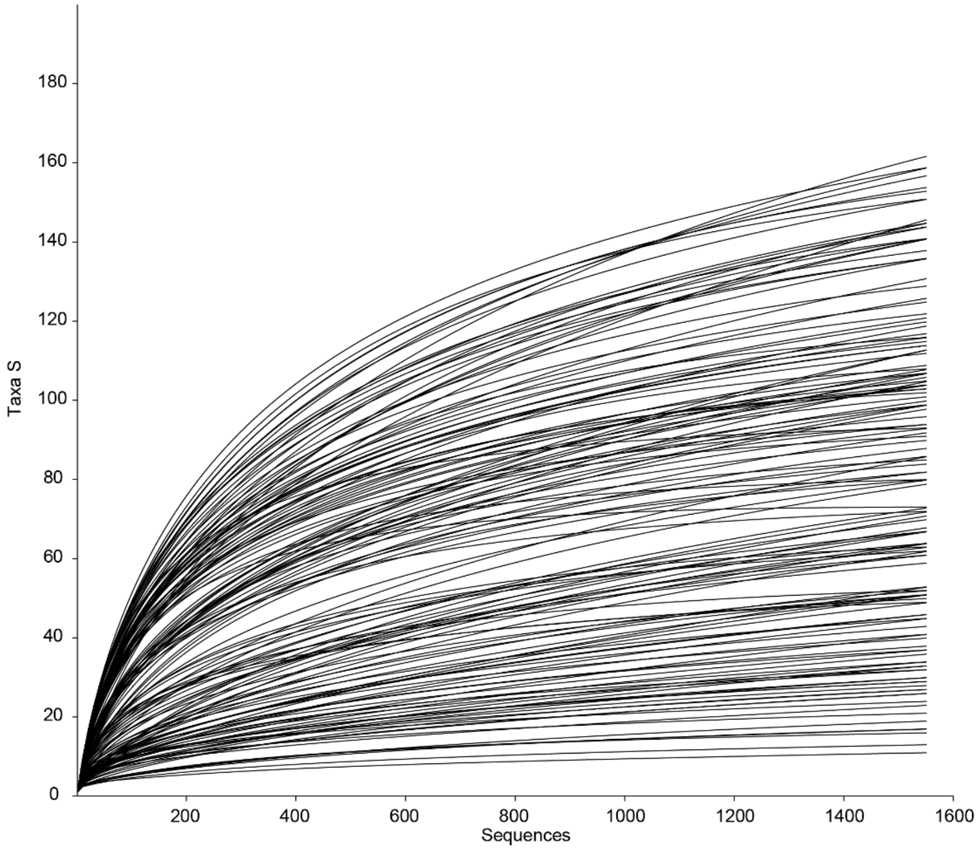

SFig 8. Rarefaction curve of the rarefied eukaryotic communities.
