# Supplementary material for: Generalist phyllosphere taxa dominate microbial communities on macrophytes across a natural salinity gradient
Source: Environ Microbiome. 2026 Apr 4;21:52. doi: 10.1186/s40793-026-00881-z (PMC13067490; doi:10.1186/s40793-026-00881-z)
Supplement: Supplementary file 2 — Supplementary Material 2. [file 40793_2026_881_MOESM2_ESM.pdf]

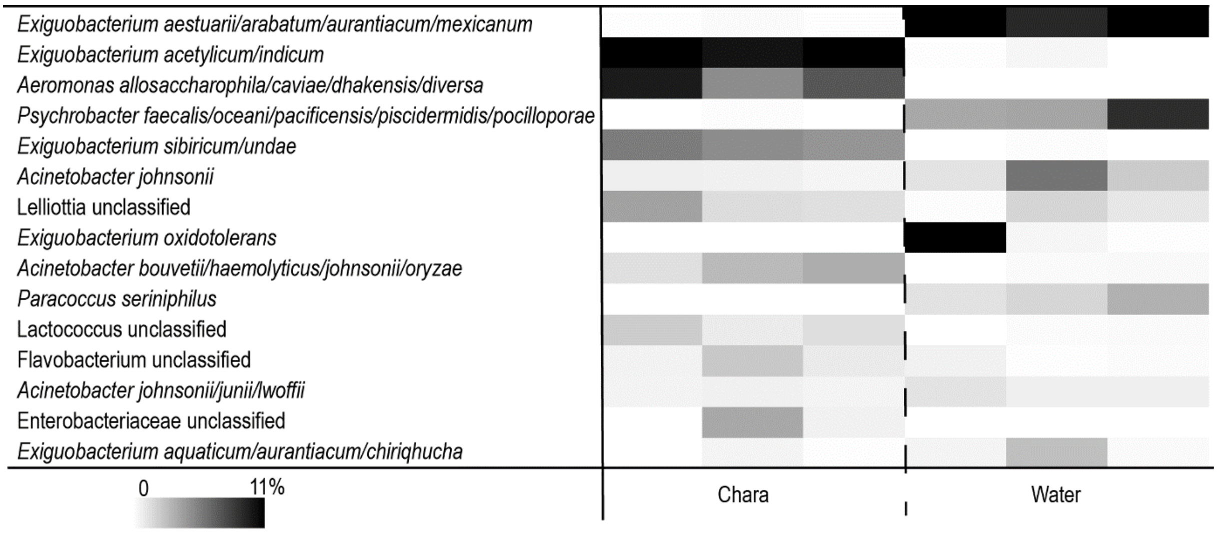

SFig 1. (A) Bacterial community composition on the Chara phyllosphere and the water phyllosphere on finest possible taxonomic resolution. ASV can be assigned to various species within a phylogenetic lineage as indicated in the different species names in the table.

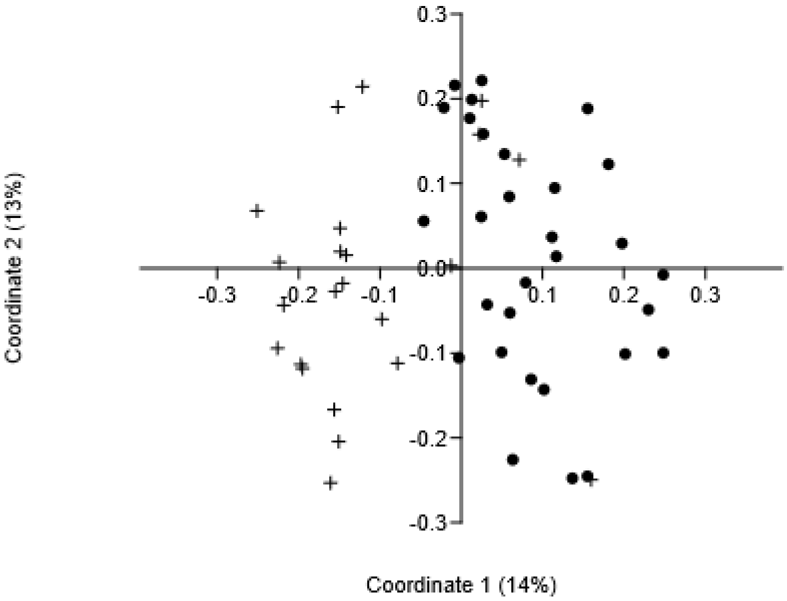

SFig 2. PCoA anlysis of alpha (dot) -and beta (plus)-mesohaline bacterial community composition on the *Zostera marina* phyllosphere.

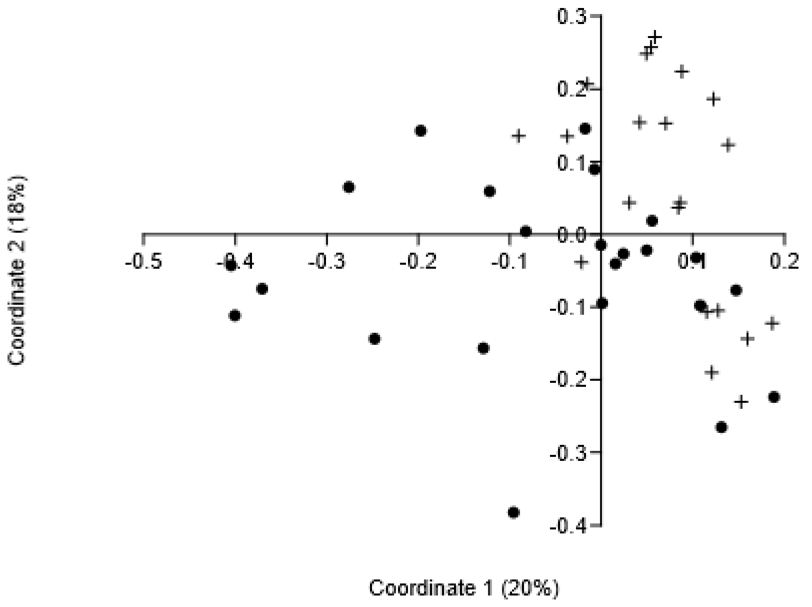

SFig 3. PCoA anlysis of Stuckneia (dot) -and *Zostera marina* (plus) phyllosphere bacterial community composition.

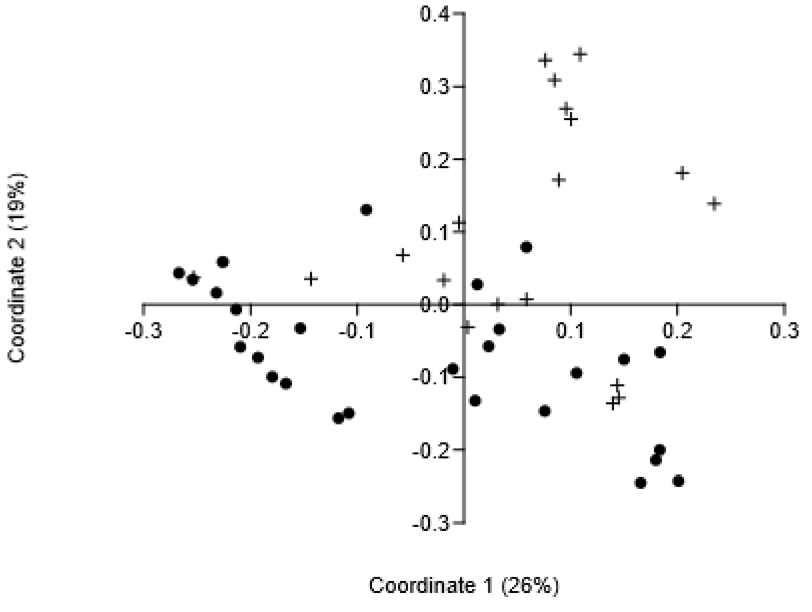

SFig 4. PCoA anlysis of alpha (dot) -and beta (plus)-mesohaline protist community composition on the *Zostera marina* phyllosphere.

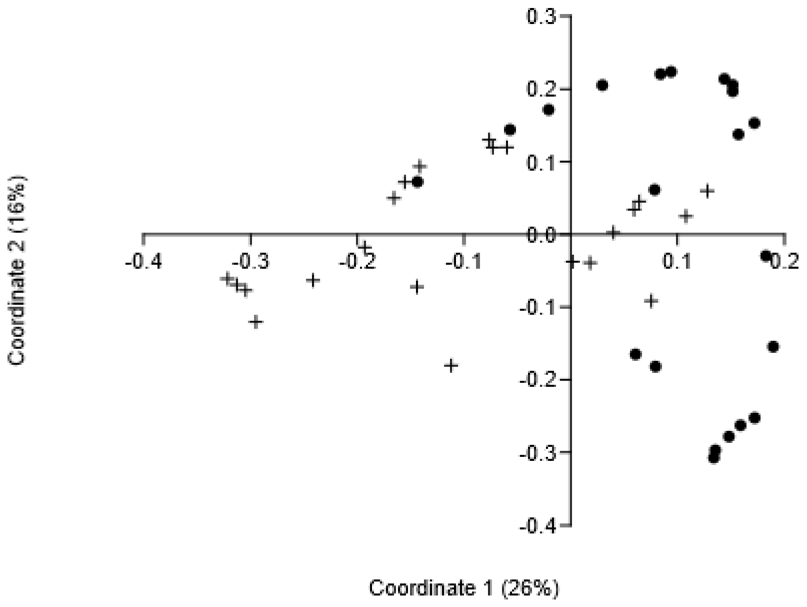

SFig 5. PCoA anlysis of Stuckneia (dot) -and *Zostera marina* (plus) phyllosphere protist community composition.

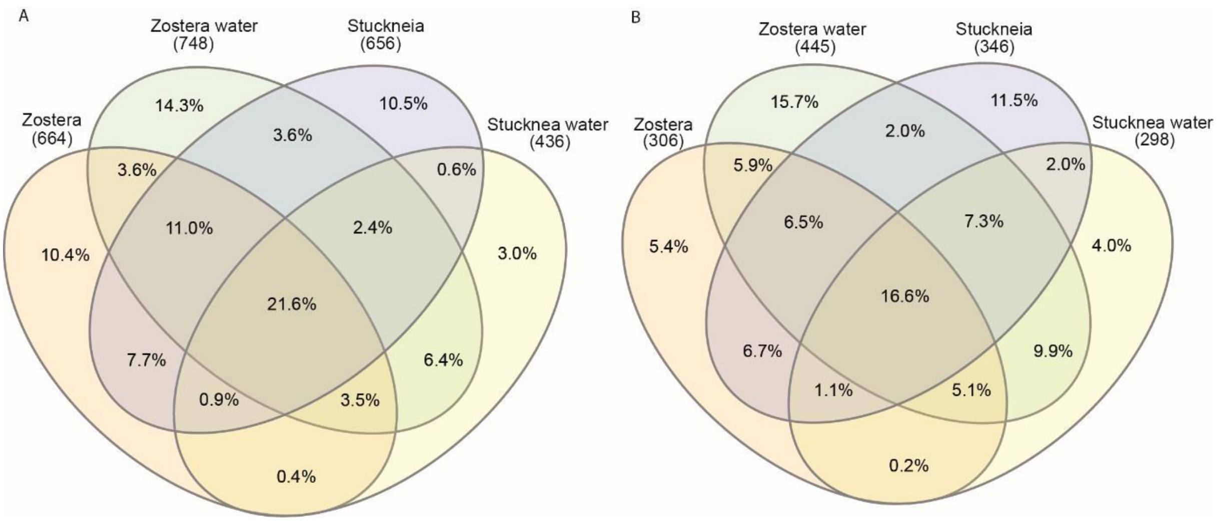

SFig 6. Venn Diagram of the (A) bacterial community and (B) microeukaryotic community composition of phyllosphere and water community at different host species. Shown are percentages of the total community.

STable 1. Result of the SIMPER Analysis comparing water and phyllosphere (PP) bacterial community

Taxon	Av. dissim	Contrib. %	Cum-ulative %	Mean PP	Mean water
p_Cyanobacteria_c_Cyanobacteriia_o_Synechococcales_f_Cyanobiaceae_g_Cyanobium_s_PCC-6307	6,061	6,528	6,528	121	1140.0
p_Proteobacteria_c_Alphaproteobacteria_o_Rhodobacterales_f_Rhodobacteraceae_	3,463	3,729	10.26	626	22.5
p_Proteobacteria_c_Gammaproteobacteria_o_Burkholderiales_f_Methylophilaceae_g_Methylotenera	3,303	3,558	13.81	580	4.17
p_Proteobacteria_c_Alphaproteobacteria_o_SAR11_f_clade_	3,198	3,444	17.26	2.26	560
p_Bacteroidota_c_Bacteroidia_o_Chitinophagales_f_Saprospiraceae_	2,905	3,129	20.39	529	24.1
p_Cyanobacteria_c_Cyanobacteriia_o_Synechococcales_f_Synechococcales_g_Incertae_s_Sedis	2,879	3.1	23.49	507	6
p_Proteobacteria_c_Alphaproteobacteria_o_SAR11_f_clade_g_Clade_s_III	2,838	3,057	26.55	2.23	497
p_Bacteroidota_c_Bacteroidia_o_Flavobacteriales_f_Flavobacteriaceae_g_NS3a_s_marine	2,791	3,006	29.55	1.21	488
p_Actinobacteriota_c_Actinobacteria_o_Frankiales_f_Sporichthyaceae_g_hgcI_s_clade	2,776	2.99	32.54	3.87	487
p_Bacteroidota_c_Bacteroidia_o_Flavobacteriales_f_Cryomorphaceae_	2,509	2,702	35.24	11.9	450
p_Proteobacteria_c_Alphaproteobacteria_o_SAR11_f_clade_g_Clade_s_I	2,394	2,578	37.82	0.811	418
p_Proteobacteria_c_Alphaproteobacteria_o_Rhodobacterales_f_Rhodobacteraceae_g_Yoonia-Loktanella	1,972	2,124	39.95	352	9.65
p_Actinobacteriota_c_Acidimicrobiia_o_Microtrichales_f_Ilumatobacteraceae_g_CL500-29_s_marine	1,367	1,472	41.42	1.51	239
p_Bacteroidota_c_Bacteroidia_o_Flavobacteriales_f_Flavobacteriaceae_g_NS5_s_marine	1,356	1,461	42.88	0.189	237
p_Proteobacteria_c_Alphaproteobacteria_o_Rhodobacterales_f_Rhodobacteraceae_g_Pseudorhodobacter	1,249	1,345	44.22	220	7.8
p_Actinobacteriota_c_Acidimicrobiia_o_Microtrichales_f_Microtrichaceae_	1,196	1,288	45.51	210	1.09
p_Proteobacteria_c_Gammaproteobacteria_o_Burkholderiales_f_Comamonadaceae_g_RS62_s_marine	1,011	1,089	46.6	2.57	179
p_Proteobacteria_c_Alphaproteobacteria_o_Rhodobacterales_f_Rhodobacteraceae_g_Planktomarina_s_temperata	0.9809	1,056	47.66	0	171
p_Proteobacteria_c_Gammaproteobacteria_o_Pseudomonadales_f_SAR86_g_clade	0.8748	0.9422	48.6	0.321	153
p_Bacteroidota_c_Bacteroidia_o_Chitinophagales_f_Saprospiraceae_g_Lewinella	0.8671	0.9339	49.53	156	6.67
p_Proteobacteria_c_Gammaproteobacteria_o_Arenicellales_f_Arenicellaceae_g_Perspicuibacter	0.8178	0.8807	50.41	142	1.5
p_Bacteroidota_c_Bacteroidia_o_Flavobacteriales_f_Flavobacteriaceae_	0.7226	0.7782	51.19	23.4	147
p_Bacteroidota_c_Bacteroidia_o_Flavobacteriales_f_Flavobacteriaceae_g_Flavobacterium	0.7036	0.7578	51.95	80.5	123
p_Bacteroidota_c_Bacteroidia_o_Chitinophagales_f_Saprospiraceae_g_Portibacter	0.686	0.7388	52.69	121	1.76
p_Proteobacteria_c_Gammaproteobacteria_o_Burkholderiales_f_MWH-UniP1_g_aquatic_s_group	0.6678	0.7192	53.41	0.189	117
p_Planctomycetota_c_Planctomycetes_o_Pirellulales_f_Pirellulaceae_	0.6422	0.6916	54.1	56.8	70.2
p_Proteobacteria_c_Gammaproteobacteria_o_Pseudomonadales_f_Pseudohongiellaceae_g_Pseudohongiella	0.6229	0.6708	54.77	1.13	110
p_Proteobacteria_c_Gammaproteobacteria_o_Pseudomonadales_f_Cellvibrionaceae_	0.6154	0.6628	55.43	110	3.26
p_Actinobacteriota_c_Actinobacteria_o_Micrococcales_f_Microbacteriaceae_g_Candidatus_s_Aquiluna	0.6141	0.6614	56.09	0.208	107
p_Proteobacteria_c_Alphaproteobacteria_o_Rhodobacterales_f_Rhodobacteraceae_g_Planktomarina	0.6046	0.6511	56.75	1.04	105
p_Proteobacteria_c_Gammaproteobacteria_o_Burkholderiales_f_Methylophilaceae_g_OM43_s_clade	0.5821	0.6269	57.37	0.0755	102
p_Proteobacteria_c_Alphaproteobacteria_o_Rhodospirillales_f_AEGEAN-169_g_marine_s_group	0.5634	0.6068	57.98	0.0943	98.3
p_Proteobacteria_c_Gammaproteobacteria_o_Burkholderiales_f_Comamonadaceae_g_Candidatus_s_Symbiobacter	0.5463	0.5884	58.57	94.9	1.76
p_Verrucomicrobiota_c_Verrucomicrobiae_o_Verrucomicrobiales_f_DEV007_	0.5327	0.5738	59.14	105	14.1
p_Bacteroidota_c_Bacteroidia_o_Sphingobacteriales_f_NS11-12_g_marine_s_group	0.5321	0.5731	59.71	19.2	107
p_Proteobacteria_c_Gammaproteobacteria_o_Granulosicoccales_f_Granulosicoccaceae_g_Granulosicoccus	0.532	0.573	60.29	94.7	2.43
p_Bacteroidota_c_Bacteroidia_o_Chitinophagales_f_Saprospiraceae_g_Rubidimonas	0.5238	0.5641	60.85	92.6	2.02
p_Proteobacteria_c_Alphaproteobacteria_o_Rhodobacterales_f_Rhodobacteraceae_g_Jannaschia	0.5123	0.5518	61.4	89.4	0.087

STable 2. Result of the SIMPER Analysis comparing alpha- and beta-mesohaline bacterial community

Taxon	Av. dissim	Contrib. %	Cumulative %	Mean alpha	Mean beta
p_Proteobacteria_c_Gammaproteobacteria_o_Burkholderiales_f_Methylophilaceae_g_Methylotenera	3,803	5,829	5,829	466	729
p_Cyanobacteria_c_Cyanobacteriia_o_Synechococcales_f_Synechococcales_g_Incertae_s_Sedis	2,952	4,525	10.35	387	665
p_Proteobacteria_c_Alphaproteobacteria_o_Rhodobacterales_f_Rhodobacteraceae_	2,783	4,265	14.62	775	431
p_Proteobacteria_c_Alphaproteobacteria_o_Rhodobacterales_f_Rhodobacteraceae_g_Pseudorhodobacter	2,497	3,828	18.45	31.8	466
p_Bacteroidota_c_Bacteroidia_o_Chitinophagales_f_Saprospiraceae_	2,331	3,573	22.02	513	551
p_Proteobacteria_c_Alphaproteobacteria_o_Rhodobacterales_f_Rhodobacteraceae_g_Yoonia-Loktanella	2	3,066	25.09	449	225
p_Proteobacteria_c_Gammaproteobacteria_o_Arenicellales_f_Arenicellaceae_g_Perspicuibacter	1.4	2,146	27.23	236	20.2
p_Actinobacteriota_c_Acidimicrobiia_o_Microtrichales_f_Microtrichaceae_	1,296	1,987	29.22	258	147
p_Cyanobacteria_c_Cyanobacteriia_o_Synechococcales_f_Cyanobiaceae_g_Cyanobium_s_PCC-6307	1,089	1,669	30.89	65.3	193
p_Proteobacteria_c_Gammaproteobacteria_o_Burkholderiales_f_Comamonadaceae_g_Candidatus_s_Symbiobacter	1,056	1,618	32.51	28.4	182
p_Proteobacteria_c_Gammaproteobacteria_o_Pseudomonadales_f_Cellvibrionaceae_	0.817	1,252	33.76	136	74.8
p_Bacteroidota_c_Bacteroidia_o_Chitinophagales_f_Saprospiraceae_g_Portibacter	0.7922	1,214	34.97	169	58.8
p_Proteobacteria_c_Gammaproteobacteria_o_Granulosicoccales_f_Granulosicoccaceae_g_Granulosicoccus	0.7561	1,159	36.13	150	23.3
p_Bacteroidota_c_Bacteroidia_o_Chitinophagales_f_Saprospiraceae_g_Rubidimonas	0.6962	1,067	37.2	86.2	101
p_Planctomycetota_c_Planctomycetes_o_Pirellulales_f_Pirellulaceae_	0.665	1,019	38.22	24.7	98.7
p_Bacteroidota_c_Bacteroidia_o_Chitinophagales_f_Saprospiraceae_g_Lewinella	0.6647	1,019	39.24	190	112
p_Proteobacteria_c_Alphaproteobacteria_o_Rhodobacterales_f_Rhodobacteraceae_g_Jannaschia	0.6555	1,005	40.24	53.2	137
p_Proteobacteria_c_Gammaproteobacteria_o_Burkholderiales_f_Comamonadaceae_g_Hydrogenophaga	0.6531	1,001	41.24	16.2	119
p_Cyanobacteria_c_Cyanobacteriia_o_Leptolyngbyales_f_Leptolyngbyaceae_	0.6442	0.9875	42.23	85.9	68.4
p_Proteobacteria_c_Alphaproteobacteria_o_Rickettsiales_f_Rickettsiaceae_g_Candidatus_s_Megaira	0.62	0.9504	43.18	87.6	70.8
p_Verrucomicrobiota_c_Verrucomicrobiae_o_Verrucomicrobiales_f_Rubritaleaceae_g_Rubritalea	0.6193	0.9492	44.13	107	2.17
p_Bacteroidota_c_Bacteroidia_o_Flavobacteriales_f_Flavobacteriaceae_g_Flavobacterium	0.6166	0.9452	45.08	58.6	109
p_Firmicutes_c_Bacilli_o_Lactobacillales_f_Streptococcaceae_g_Streptococcus_s_anginosus	0.5868	0.8994	45.97	102	0
p_Bacteroidota_c_Bacteroidia_o_Flavobacteriales_f_Flavobacteriaceae_g_Algibacter	0.5635	0.8638	46.84	98.2	0.261
p_Bacteroidota_c_Bacteroidia_o_Cytophagales_f_Amoebophilaceae_g_Candidatus_s_Amoebophilus	0.5551	0.8508	47.69	2.5	98.5
p_Proteobacteria_c_Alphaproteobacteria_o_Rhodobacterales_f_Rhodobacteraceae_g_Pacificibacter_s_marinus	0.5498	0.8427	48.53	76.9	51.5
p_Bacteroidota_c_Bacteroidia_o_Flavobacteriales_f_Flavobacteriaceae_g_Maribacter	0.5232	0.8019	49.33	23.9	101
p_Verrucomicrobiota_c_Verrucomicrobiae_o_Verrucomicrobiales_f_DEV007_	0.5122	0.7852	50.12	120	84.9

STable 3. Result of the SIMPER Analysis comparing Stuckneia and Zostera marina bacterial community

Taxon	Av. dissim	Contrib. %	Cumulative %	Mean Stuckenia	Mean Zostera
p_Proteobacteria_c_Alphaproteobacteria_o_Rhodobacterales_f_Rhodobacteraceae_g_Pseudorhodobacter	1,773	3,241	18.79	207	466
p_Bacteroidota_c_Bacteroidia_o_Chitinophagales_f_Saprospiraceae_	1,672	3,055	21.85	529	551
p_Cyanobacteria_c_Cyanobacteriia_o_Synechococcales_f_Cyanobiaceae_g_Cyanobium_s_PCC-6307	1,494	2,731	24.58	238	193
p_Proteobacteria_c_Alphaproteobacteria_o_Rhodobacterales_f_Rhodobacteraceae_	1,302	2,379	26.96	291	431
p_Proteobacteria_c_Alphaproteobacteria_o_Rhodobacterales_f_Rhodobacteraceae_g_Yoonia-Loktanella	1,146	2,094	29.05	53	225
p_Actinobacteriota_c_Acidimicrobiia_o_Microtrichales_f_Microtrichaceae_	1,059	1,936	30.99	179	147
p_Proteobacteria_c_Gammaproteobacteria_o_Burkholderiales_f_Comamonadaceae_g_Candidatus_s_Symbiobacter	1,026	1,875	32.86	8.95	182
p_Proteobacteria_c_Alphaproteobacteria_o_Rhodobacterales_f_Rhodobacteraceae_g_Jannaschia	1,021	1,867	34.73	230	137
p_Cyanobacteria_c_Cyanobacteriia_o_Synechococcales_f_Synechococcales_g_Incertae_s_Sedis	5.1	9,319	9,319	1340	665
p_Proteobacteria_c_Gammaproteobacteria_o_Burkholderiales_f_Methylophilaceae_g_Methylotenera	3.41	6,232	15.55	501	729
p_Bacteroidota_c_Bacteroidia_o_Chitinophagales_f_Saprospiraceae_g_Rubidimonas	0.8054	1,472	36.2	147	101
p_Cyanobacteria_c_Cyanobacteriia_o_Phormidesmiales_f_Phormidesmiaceae_g_Phormidesmis_s_ANT.LACV5.1	0.7683	1,404	37.6	192	67.4
p_Bacteroidota_c_Bacteroidia_o_Flavobacteriales_f_Flavobacteriaceae_g_Maribacter	0.6538	1,195	38.8	166	101
p_Bacteroidota_c_Bacteroidia_o_Flavobacteriales_f_Flavobacteriaceae_g_Flavobacterium	0.6039	1,104	39.9	83.1	109
p_Proteobacteria_c_Gammaproteobacteria_o_Burkholderiales_f_Comamonadaceae_g_Hydrogenophaga	0.6032	1,102	41.01	108	119
p_Chloroflexi_c_Anaerolineae_o_Caldilineales_f_Caldilineaceae_	0.5867	1,072	42.08	128	40.9
p_Planctomycetota_c_Planctomycetes_o_Pirellulales_f_Pirellulaceae_	0.5823	1,064	43.14	9.67	98.7
p_Bacteroidota_c_Bacteroidia_o_Cytophagales_f_Amoebophilaceae_g_Candidatus_s_Amoebophilus	0.5337	0.9753	44.12	60.8	98.5

STable 4. Result of the SIMPER Analysis comparing water and phyllosphere (PP) protist community

Taxon	Av. dissim	Contrib. %	Cumulative %	Mean PP	Mean water
TSAR;Stramenopiles;Gyrista;Phaeophyceae;Phaeophyceae_X;Phaeophyceae_XX;Phaeophyceae_XXX;Phaeophyceae_XXX_sp.	11.87	12.17	12.17	373	3.18
TSAR;Stramenopiles;Gyrista;Bacillariophyceae	6,981	7,157	19.32	219	1.68
TSAR;Stramenopiles;Gyrista;Bacillariophyceae;Achnanthales;Cocconeidaceae;Cocconeis;Cocconeis_placentula	6.83	7,002	26.33	213	0.31
TSAR;Stramenopiles;Gyrista;Bacillariophyceae;Achnanthales	4,369	4,479	30.81	136	0.0141
TSAR;Alveolata;Ciliophora;Heterotrichea;Heterotrichea_X;Folliculinidae;Folliculinidae_X;Folliculinidae_X_sp.	3,126	3,204	34.01	97.6	0.141
TSAR;Stramenopiles;Gyrista;Coscinodiscophyceae;Rhizosoleniales;Rhizosoleniaceae;Rhizosolenia;R.delicatula	2,352	2,411	36.42	0.027	73.4
Archaeplastida;Chlorophyta;Chlorophyta_X;Ulvophyceae;Ulvales-relatives;Ulvales-relatives_X;Acrochaete;A.leptochaete	1,956	2,005	38.43	61.1	1.79
TSAR;Stramenopiles;Gyrista;Mediophyceae;Thalassiosirales;Stephanodiscaceae;Cyclotella	1,689	1,731	40.16	0.459	53
Archaeplastida;Chlorophyta;Chlorophyta_X;Chlorophyceae;Sphaeropleales;Sphaeropleales_X	1,662	1,704	41.86	23.2	35.7
Cryptista;Cryptophyta;Cryptophyta_X;Cryptophyceae;Cryptomonadales;Cryptomonadales_X;Plagioselmis;P.prolonga	1,561	1.6	43.46	0.0541	48.8
Cryptista;Cryptophyta;Cryptophyta_X;Cryptophyceae;Cryptomonadales;Cryptomonadales_X;Falcomonas;F.daucoides	1,539	1,578	45.04	0.0541	48.1
Archaeplastida;Chlorophyta;Chlorophyta_X;Trebouxiophyceae;Chlorellales;Chlorellales_X;Picochlorum;Picochlorum_sp.	1,501	1,539	46.58	2.86	47.9
Archaeplastida;Chlorophyta;Chlorophyta_X;Mamiellophyceae;Mamiellales;Bathycoccaceae;Ostreococcus;O.tauri	1,467	1,504	48.08	0.0405	45.8
Archaeplastida;Chlorophyta;Chlorophyta_X;Pedinophyceae;Marsupiomonadales;Marsupiomonadaceae;Marsupiomonas	1,231	1,262	49.34	0.108	38.5
Archaeplastida;Chlorophyta;Chlorophyta_X;Chlorophyceae;Sphaeropleales;Sphaeropleales_X;Desmodesmus	1,124	1,152	50.5	11.2	25.6
Archaeplastida;Chlorophyta;Chlorophyta_X;Mamiellophyceae;Mamiellales;Mamiellaceae;Micromonas;M.bravo_B2	1,105	1,133	51.63	0.027	34.5
TSAR;Stramenopiles;Gyrista;Bacillariophyceae;Achnanthales;Cocconeidaceae;Cocconeis	1,045	1,071	52.7	32.7	0.183
TSAR;Stramenopiles;Gyrista;Phaeophyceae;Phaeophyceae_X;Phaeophyceae_XX	1,004	1,029	53.73	31.6	1.13
Archaeplastida;Chlorophyta;Chlorophyta_X;Trebouxiophyceae;Trebouxiophyceae_X;Trebouxiophyceae_XX;Choricystis	0.9273	0.9507	54.68	1.91	30.4
TSAR;Stramenopiles;Gyrista;Eustigmatophyceae;Eustigmatophyceae_X;Eustigmatophyceae_XX;Monodus;Monodus_sp.	0.8759	0.898	55.58	7.01	23.4
TSAR;Rhizaria;Cercozoa;Filosa-Thecofilosea;Cryomonadida;Protaspa-lineage;Protaspa-lineage_X	0.862	0.8837	56.46	1.11	27.8
TSAR;Stramenopiles;Gyrista;Mediophyceae;Thalassiosirales;Thalassiosiraceae;Thalassiosira;T._pseudonana	0.8286	0.8495	57.31	0.649	25.9
Archaeplastida;Rhodophyta;Rhodophyta_X;Florideophyceae;Ceramiales;Ceramiaceae;Ceramium	0.814	0.8345	58.15	20	7.15
TSAR;Alveolata;Dinoflagellata;Syndiniales;Dino-Group-I;Dino-Group-I-Clade-4;Dino-Group-I-Clade-4_X	0.8027	0.823	58.97	0.932	25.3
TSAR;Alveolata;Dinoflagellata;Dinophyceae;Peridiniales;Heterocapsaceae;Heterocapsa;Heterocapsa_pygmaea	0.7629	0.7821	59.75	0.365	23.9
Archaeplastida;Rhodophyta;Rhodophyta_X;Florideophyceae;Acrochaetiales;Acrochaetiales_X;Acrochaetiales_XX	0.7309	0.7493	60.5	19.8	3.69
TSAR;Stramenopiles;Gyrista;Mediophyceae;Chaetocerotales;Chaetocerotaceae;Chaetoceros;Chaetoceros_pumilum	0.6827	0.6999	61.2	0.0541	21.3
Archaeplastida;Chlorophyta;Chlorophyta_X;Chlorophyceae;Sphaeropleales;Sphaeropleales_X;Scenedesmus;S.armatus	0.6776	0.6947	61.89	6.2	16.5
Archaeplastida;Chlorophyta;Chlorophyta_X;Chlorophyceae;Sphaeropleales;Sphaeropleales_X;Desmodesmus;D.pannonicus	0.6648	0.6816	62.58	7.8	14.6
Archaeplastida;Chlorophyta;Chlorophyta_X;Chlorophyceae;Chlamydomonadales;Chlamydomonadales_X	0.6536	0.6701	63.25	0.554	20.6
TSAR;Stramenopiles;Gyrista;Phaeophyceae;Phaeophyceae_X;Phaeophyceae_XX;Ectocarpus;Ectocarpus_siliculosus	0.6523	0.6687	63.91	18.6	2.31
TSAR;Alveolata;Ciliophora;Spirotrichea;Oligotrichida;Strombidiidae;Strombidium;Strombidium_paracapitatum	0.6382	0.6543	64.57	0.0811	20
TSAR;Stramenopiles;Gyrista;Gyrista_X;Gyrista_XX;MAST-1;MAST-1C;MAST-1C_sp.	0.6379	0.6539	65.22	0.149	20
Archaeplastida;Chlorophyta;Chlorophyta_X;Trebouxiophyceae;Chlorellales;Chlorellales_X	0.6358	0.6518	65.87	0.527	20
Cryptista;Cryptophyta;Cryptophyta_X;Cryptophyceae;Cryptomonadales;Cryptomonadales_X;Teleaulax;Teleaulax_acuta	0.6147	0.6302	66.5	0.0541	19.2
Obazoa;Opisthokonta	0.5939	0.6089	67.11	3.15	17.8
Archaeplastida;Rhodophyta;Rhodophyta_X;Florideophyceae;Ceramiales;Callithamniaceae;Callithamnion;C.collabens	0.5753	0.5898	67.7	0.0405	17.9
TSAR;Alveolata;Dinoflagellata;Dinophyceae	0.558	0.572	68.28	1.89	17.2

STable 5. Result of the SIMPER Analysis comparing alpha- and beta-mesohaline protist community

Taxon	Av. dissim	Contrib. %	Cumulative %	Mean alpha	Mean beta
TSAR;Stramenopiles;Gyrista;Phaeophyceae;Phaeophyceae_X;Phaeophyceae_XX;Phaeophyceae_XXX;Phaeophyceae_XXX_sp.	14.82	21.72	21.72	576	340
TSAR;Stramenopiles;Gyrista;Bacillariophyceae	10.47	15.34	37.07	303	312
TSAR;Stramenopiles;Gyrista;Bacillariophyceae;Achnanthales;Cocconeidaceae;Cocconeis;Cocconeis_placentula	9,107	13.35	50.42	8.52	291
TSAR;Stramenopiles;Gyrista;Bacillariophyceae;Achnanthales	8,759	12.84	63.25	276	113
TSAR;Alveolata;Ciliophora;Heterotrichea;Heterotrichea_X;Folliculinidae;Folliculinidae_X;Folliculinidae_X_sp.	2,906	4,259	67.51	21.3	83.5
TSAR;Stramenopiles;Gyrista;Phaeophyceae;Phaeophyceae_X;Phaeophyceae_XX;Ectocarpus;Ectocarpus_siliculosus	2,075	3,042	70.56	3.04	62.9
TSAR;Stramenopiles;Gyrista;Phaeophyceae;Phaeophyceae_X;Phaeophyceae_XX	1,749	2,564	73.12	20.9	60.3
Archaeplastida;Rhodophyta;Rhodophyta_X;Florideophyceae;Acrochaetiales;Acrochaetiales_X;Acrochaetiales_XX	1.68	2,462	75.58	51.9	2.58
TSAR;Stramenopiles;Gyrista;Bacillariophyceae;Achnanthales;Cocconeidaceae;Cocconeis	1,596	2,339	77.92	28.4	58.3
TSAR;Alveolata;Ciliophora;Oligohymenophorea;Peritrichia_2;Sessilida;Pseudovorticella	0.8245	1,208	79.13	25.7	0
Archaeplastida;Chlorophyta;Chlorophyta_X;Ulvophyceae;Ulvales-relatives;Ulvales-relatives_X;Acrochaete;A.leptochaete	0.6519	0.9554	80.08	3.48	21.3
TSAR;Stramenopiles;Gyrista;Eustigmatophyceae;Eustigmatophyceae_X;Eustigmatophyceae_XX;Monodus;Monodus_sp.	0.5933	0.8697	80.95	0.778	18.1
Obazoa;Opisthokonta;Choanoflagellata;Choanoflagellatea	0.5074	0.7437	81.7	14	11.1
TSAR;Stramenopiles;Gyrista;Bacillariophyceae;Licmophorales;Ulnariaceae;Tabularia	0.4833	0.7083	82.41	7	10.4
TSAR;Alveolata;Ciliophora;Oligohymenophorea;Peritrichia_2;Sessilida;Vorticella	0.4816	0.7059	83.11	0.296	14.8
TSAR;Stramenopiles;Gyrista;Bacillariophyceae;Achnanthales;Cocconeidaceae;Cocconeis;Cocconeis_pediculus	0.4703	0.6894	83.8	0	14.7
TSAR;Alveolata;Ciliophora;Oligohymenophorea;Peritrichia_2;Sessilida;Pseudovorticella;Pseudovorticella_paracratera	0.4516	0.6619	84.46	14.1	0.0526
TSAR;Stramenopiles;Gyrista;Bacillariophyceae;Naviculales;Berkeleyaceae;Berkeleya;Berkeleya_hyalina	0.4057	0.5947	85.06	12.7	0
TSAR;Rhizaria;Cercozoa;Endomyxa;Vampyrellida;op14-lineage;op14-lineage_X;op14-lineage_X_sp.	0.3286	0.4816	85.54	10.3	0.316
TSAR;Stramenopiles;Bigyra;Bicoecea;Bicoecales;Bicoecaceae;Bicosoeca	0.3063	0.4489	85.99	5.59	4.95
TSAR;Stramenopiles;Gyrista;Bacillariophyceae;Rhabdonematales;Grammatophoraceae;Grammatophora;G.oceanica	0.2982	0.4371	86.43	9.19	0.316
TSAR;Stramenopiles;Gyrista;Coscinodiscophyceae;Melosirales;Melosiraceae;Melosira	0.2761	0.4047	86.83	0.481	8.26
TSAR;Stramenopiles;Gyrista;Peronosporomycetes;Peronosporomycetes_X;Anisolpidium;A.rosenvingei	0.273	0.4001	87.23	5.48	3.89
TSAR;Rhizaria;Cercozoa;Endomyxa;Vampyrellida;Vampyrellida_X;Vampyrellida_XX;Vampyrellida_XX_sp.	0.2712	0.3974	87.63	7.63	1.63
Obazoa;Opisthokonta;Fungi;Chytridiomycota	0.2397	0.3513	87.98	7.3	2.11
TSAR;Alveolata;Ciliophora;Oligohymenophorea;Peritrichia_2;Sessilida	0.2331	0.3416	88.32	2.7	5.58
TSAR;Stramenopiles;Gyrista;Bacillariophyceae;Naviculales;Naviculaceae;Navicula	0.2147	0.3147	88.64	3.59	4.21
TSAR;Stramenopiles;Gyrista	0.2013	0.295	88.93	6.07	0.526
Archaeplastida;Chlorophyta;Chlorophyta_X;Trebouxiophyceae;Chlorellales;Chlorellales_X;Picochlorum;Picochlorum_sp.	0.2011	0.2947	89.23	0.481	6.16
Archaeplastida;Chlorophyta;Chlorophyta_X;Chlorophyceae;Sphaeropleales;Sphaeropleales_X	0.1871	0.2742	89.5	1.07	5.42
TSAR;Stramenopiles;Gyrista;Bacillariophyceae;Licmophorales;Licmophoraceae;Licmophora;Licmophora_sp.	0.1555	0.2279	89.73	4.78	0.158
TSAR;Stramenopiles;Bigyra;Sagenista;Labyrinthulomycetes;Thraustochytriaceae;Thraustochytriaceae_X	0.151	0.2213	89.95	4.7	0.0526
Obazoa;Opisthokonta;Fungi;Chytridiomycota;Rhizophydiales	0.1424	0.2087	90.16	4.37	0.105
Archaeplastida;Chlorophyta;Chlorophyta_X;Chlorophyceae;Oedogoniales;Oedogoniales_X;Oedogonium;Oedogonium_sp.	0.1298	0.1903	90.35	0	4.05
TSAR;Alveolata;Dinoflagellata;Dinophyceae;Gymnodiniales;Gymnodiniaceae;Amphidinium	0.1293	0.1895	90.54	4.04	0
TSAR;Stramenopiles;Gyrista;Coscinodiscophyceae;Melosirales;Melosiraceae;Melosira;Melosira_sp.	0.1277	0.1872	90.72	0.148	3.89
TSAR;Rhizaria;Cercozoa;Endomyxa;Endomyxa_X;Endomyxa_Novel-clade-9;Endomyxa_Novel-clade-9_X	0.1236	0.1811	90.91	1.33	3.74
Archaeplastida;Chlorophyta;Chlorophyta_X;Ulvophyceae;Ulvales-relatives;Ulvales-relatives_X;Ulva;Ulva_laetevirens	0.1211	0.1774	91.08	2.07	2.37

TSAR;Stramenopiles;Bigyra;Opalozoa;Nanomonadea;MAST-3;MAST-3J;MAST-3J_sp.	0.1201	0.1761	91.26	3.74	0.158
Haptista;Centroplasthelida;Centroplasthelida_X;Centroplasthelida_XX;Centroplasthelida_XXX;Centroplasthelida_XXXX;	0.1056	0.1548	91.41	3.15	0.526
TSAR;Stramenopiles;Gyrista;Coscinodiscophyceae;Melosirales;Hyalodiscaceae;Podosira;Podosira_stelligera	0.1044	0.153	91.57	3.26	0
TSAR;Stramenopiles;Gyrista;Bacillariophyceae;Naviculales;Naviculaceae;Navicula;Navicula_sp.	0.09984	0.1463	91.71	3.15	0.316
TSAR;Stramenopiles;Gyrista;Peronosporomycetes;Peronosporomycetes_X	0.09815	0.1439	91.86	2.44	1.05
TSAR;Stramenopiles;Bigyra;Sagenista;Labyrinthulomycetes;Labyrinthulaceae;Labyrinthula;Labyrinthula_sp.	0.09722	0.1425	92	2.67	0.474
TSAR;Alveolata;Ciliophora;Spirotrichea;Euplotia;Aspidiscidae;Aspidisca;Aspidisca_aculeata	0.09316	0.1365	92.14	2.89	0.105
TSAR;Alveolata;Dinoflagellata;Syndiniales;Dino-Group-I;Dino-Group-I-Clade-4;Dino-Group-I-Clade-4_X	0.0856	0.1255	92.26	0.0741	2.68
TSAR;Stramenopiles;Gyrista;Bacillariophyceae;Achnanthales;Achnanthidiaceae	0.08279	0.1213	92.38	2.52	0.211
TSAR;Alveolata;Ciliophora;Oligohymenophorea;Peritrichia_2;Sessilida;Planeticovorticella;Planeticovorticella_paradoxa	0.08167	0.1197	92.5	0.148	2.53
TSAR;Stramenopiles;Bigyra;Sagenista;Labyrinthulomycetes;Oblongichytridiaceae;Oblongichytrium;Oblongichytrium_sp.	0.08129	0.1192	92.62	1.81	0.895
Obazoa;Opisthokonta;Choanoflagellata;Choanoflagellatea;Craspedida;Salpingoecidae_Group_C2	0.08117	0.119	92.74	2.44	0.526
TSAR;Alveolata;Ciliophora;Oligohymenophorea;Peritrichia_2;Sessilida;Zoothamnium_1;Zoothamnium_bucciniiformum	0.08098	0.1187	92.86	0.0741	2.47
TSAR;Stramenopiles;Gyrista;Mediophyceae;Thalassiosirales;Stephanodiscaceae;Discostella;Discostella_sp.	0.0793	0.1162	92.98	1.04	1.58
TSAR;Stramenopiles;Gyrista;Bacillariophyceae;Rhopalodiales;Rhopalodiaceae;Epithemia;Epithemia_turgida	0.07699	0.1128	93.09	0.0741	2.37
TSAR;Stramenopiles;Gyrista;Bacillariophyceae;Naviculales;Naviculaceae	0.07574	0.111	93.2	2.3	0.158
TSAR;Stramenopiles;Gyrista;Bacillariophyceae;Mastogloiales;Mastogloiaceae;Mastogloia;Mastogloia_sp.	0.07511	0.1101	93.31	2.26	0.211
TSAR;Alveolata;Ciliophora;Heterotrichea;Heterotrichea_X;Stentoridae;Stentor;Stentor_muelleri	0.07499	0.1099	93.42	0.037	2.32
TSAR;Alveolata;Ciliophora;Oligohymenophorea;Peritrichia_2;Sessilida;Zoothamnium_1;Zoothamnium_grossi	0.07411	0.1086	93.53	0.333	2.11
TSAR;Stramenopiles;Gyrista;Bacillariophyceae;Thalassiophysales;Catenulaceae;Amphora	0.07312	0.1072	93.64	1.67	1.05
TSAR;Stramenopiles;Gyrista;Bacillariophyceae;Bacillariales;Bacillariaceae;Nitzschia;Nitzschia_inconspicua	0.06824	0.1	93.74	1.96	0.316
Obazoa;Opisthokonta;Fungi;Chytridiomycota;Chytridiales;Chytridiaceae;Chytridium;Chytridium_polysiphoniae	0.06606	0.09682	93.83	0.037	2.05
Archaeplastida;Chlorophyta;Chlorophyta_X;Trebouxiophyceae;Trebouxiophyceae_X;Trebouxiophyceae_XX;Choricystis	0.06456	0.09463	93.93	0.519	1.89
TSAR;Alveolata;Ciliophora;Oligohymenophorea;Peritrichia_2;Sessilida;Zoothamnium_1	0.06263	0.09179	94.02	0.963	1.21
TSAR;Stramenopiles	0.06113	0.08959	94.11	1.89	0.105
TSAR;Alveolata;Ciliophora;Spirotrichea;Hypotrichia	0.05938	0.08703	94.2	1.56	0.474
Archaeplastida;Chlorophyta;Chlorophyta_X;Chlorophyceae;Chaetopeltidales;Chaetopeltis;C._orbicularis	0.059	0.08648	94.28	0	1.84
TSAR;Alveolata;Ciliophora;Spirotrichea;Hypotrichia;Holostichidae;Holosticha;Holosticha_diademata	0.05875	0.08612	94.37	0.481	1.58
Archaeplastida;Chlorophyta;Chlorophyta_X;Chlorodendrophyceae;Chlorodendrales;Chlorodendraceae;Chlorodendrales_XX	0.05576	0.08172	94.45	1.74	0
Obazoa;Opisthokonta;Fungi	0.05576	0.08172	94.53	0.852	1.26
TSAR;Stramenopiles;Gyrista;Bacillariophyceae;Naviculales;Naviculaceae;Navicula;Navicula_perminuta	0.0552	0.0809	94.61	1.67	0.684
TSAR;Alveolata;Ciliophora;Oligohymenophorea;Peritrichia_2;Sessilida;Sessilida_X;Sessilida_X_sp.	0.05189	0.07605	94.69	0.444	1.42
TSAR;Stramenopiles;Bigyra;Sagenista;Labyrinthulomycetes;Labyrinthulaceae;Aplanochytrium;Aplanochytrium_sp.	0.05182	0.07596	94.76	0.222	1.58
TSAR;Stramenopiles;Gyrista;Peronosporomycetes;Peronosporomycetes_X;Peronosporomycetes_XX;Peronosporomycetes_XXX	0.05114	0.07495	94.84	1.48	0.158
TSAR;Stramenopiles;Gyrista;Bacillariophyceae;Achnanthales;Cocconeidaceae;Cocconeis;Cocconeis_stauroneiformis	0.05101	0.07477	94.91	1.59	0

Stable 6. Result of the SIMPER Analysis comparing Stuckneia and Zostera marina bacterial community

Taxon	Av. dissim	Contrib. %	Cumulative %	Mean Stuckenia	Mean Zostera
TSAR;Stramenopiles;Gyrista;Bacillariophyceae;Achnanthales;Cocconeidaceae;Cocconeis;Cocconeis_placentula	9,426	11.18	11.18	289	130
TSAR;Stramenopiles;Gyrista;Phaeophyceae;Phaeophyceae_X;Phaeophyceae_XX;Phaeophyceae_XXX;Phaeophyceae_XXX_sp.	7,989	9,478	20.66	54.9	239
TSAR;Alveolata;Ciliophora;Heterotrichea;Heterotrichea_X;Folliculinidae;Folliculinidae_X;Folliculinidae_X_sp.	5,628	6,677	27.34	153	37.1
TSAR;Stramenopiles;Gyrista;Bacillariophyceae	5,166	6,129	33.47	50.1	146
Archaeplastida;Chlorophyta;Chlorophyta_X;Ulvophyceae;Ulvales-relatives;Ulvales-relatives_X;Acrochaete;Acrochaete_leptochaete	3,967	4,706	38.17	123	10.6
TSAR;Stramenopiles;Gyrista;Bacillariophyceae;Achnanthales	1,902	2,257	40.43	14.7	50.1
TSAR;Stramenopiles;Gyrista;Mediophyceae;Thalassiosirales;Stephanodiscaceae;Cyclotella	1,617	1,918	42.35	18.6	44.9
Archaeplastida;Chlorophyta;Chlorophyta_X;Trebouxiophyceae;Chlorellales;Chlorellales_X;Picochlorum;Picochlorum_sp.	1,608	1,907	44.25	12.9	46.8
Cryptista;Cryptophyta;Cryptophyta_X;Cryptophyceae;Cryptomonadales;Cryptomonadales_X;Falcomonas;F._daucoides	1,537	1,823	46.08	32.4	29.4
TSAR;Stramenopiles;Gyrista;Mediophyceae;Thalassiosirales;Thalassiosiraceae;Thalassiosira;T._pseudonana	1,508	1,789	47.87	46.6	4.7
Archaeplastida;Rhodophyta;Rhodophyta_X;Florideophyceae;Ceramiales;Ceramiaceae;Ceramium	1,455	1,727	49.59	45.7	1.3
Cryptista;Cryptophyta;Cryptophyta_X;Cryptophyceae;Cryptomonadales;Cryptomonadales_X;Plagioselmis;P.prolonga	1.13	1.34	50.93	23.4	25
TSAR;Stramenopiles;Gyrista;Phaeophyceae;Phaeophyceae_X;Phaeophyceae_XX	1,117	1,325	52.26	19	27.3
TSAR;Alveolata;Dinoflagellata;Syndiniales;Dino-Group-I;Dino-Group-I-Clade-4;Dino-Group-I-Clade-4_X	1,086	1,289	53.55	3.52	33.8
TSAR;Stramenopiles;Gyrista;Phaeophyceae;Phaeophyceae_X;Phaeophyceae_XX;Ectocarpus;Ectocarpus_siliculosus	1,062	1.26	54.81	3.42	30.7
Archaeplastida;Chlorophyta;Chlorophyta_X;Chlorophyceae;Sphaeropleales;Sphaeropleales_X	1,053	1,249	56.06	29.3	8.6
TSAR;Stramenopiles;Gyrista;Eustigmatophyceae;Eustigmatophyceae_X;Eustigmatophyceae_XX;Monodus;Monodus_sp.	1,008	1,196	57.25	3.97	30.4
Archaeplastida;Chlorophyta;Chlorophyta_X;Pedinophyceae;Marsupiomonadales;Marsupiomonadaceae;Marsupiomonas	0.9986	1,185	58.44	7.7	29.1
TSAR;Stramenopiles;Gyrista;Bacillariophyceae;Achnanthales;Cocconeidaceae;Cocconeis	0.9722	1,153	59.59	16.3	25.9
TSAR;Rhizaria;Cercozoa;Filosa-Thecofilosea;Cryomonadida;Protaspa-lineage;Protaspa-lineage_X	0.8917	1,058	60.65	22.8	17
Obazoa;Opisthokonta	0.8829	1,047	61.7	13.8	20
TSAR;Stramenopiles;Gyrista;Mediophyceae;Chaetocerotales;Chaetocerotaceae;Chaetoceros;Chaetoceros_pumilum	0.8162	0.9683	62.66	14	16.1
TSAR;Alveolata;Ciliophora;Oligohymenophorea;Peritrichia_2;Sessilida;Vorticella	0.7801	0.9256	63.59	17.7	9.58
Archaeplastida;Chlorophyta;Chlorophyta_X;Chlorophyceae;Chlamydomonadales;Chlamydomonadales_X	0.7778	0.9228	64.51	18	12.3
TSAR;Alveolata;Ciliophora;Spirotrichea;Oligotrichida;Strombidiidae;Strombidium;Strombidium_paracapitatum	0.7291	0.865	65.38	12	14.5
TSAR;Stramenopiles;Gyrista;Coscinodiscophyceae;Melosirales;Melosiraceae;Melosira;Melosira_sp.	0.6189	0.7342	66.11	17.9	2.51
Cryptista;Cryptophyta;Cryptophyta_X;Cryptophyceae;Cryptomonadales;Cryptomonadales_X;Teleaulax;Teleaulax_acuta	0.6063	0.7193	66.83	8.97	15.2
Archaeplastida;Chlorophyta;Chlorophyta_X;Trebouxiophyceae;Trebouxiophyceae_X;Trebouxiophyceae_XX;Choricystis	0.5778	0.6855	67.52	13.9	14.2
TSAR;Stramenopiles;Gyrista;Gyrista_X;Gyrista_XX;MAST-1;MAST-1C;MAST-1C_sp.	0.576	0.6834	68.2	16.5	3.77
TSAR;Rhizaria;Cercozoa;Filosa-Thecofilosea;Ebriacea;Ebriidae;Ebria;Ebria_tripartita	0.5717	0.6783	68.88	12.1	9.95
Archaeplastida;Chlorophyta;Chlorophyta_X;Chlorophyceae;Chlamydomonadales;Chlamydomonadales_X	0.5303	0.6292	69.51	8.45	11.6
Obazoa;Opisthokonta;Choanoflagellata;Choanoflagellatea	0.5289	0.6275	70.13	16.6	4.91
Archaeplastida;Chlorophyta;Chlorophyta_X;Mamiellophyceae;Mamiellales;Bathycoccaceae;Ostreococcus;O.tauri	0.5068	0.6012	70.74	0.727	15.6
